# An Autonomous Self-Aware and Adaptive Fault Tolerant Routing Technique for Wireless Sensor Networks

**DOI:** 10.3390/s150820316

**Published:** 2015-08-18

**Authors:** Sani Abba, Jeong-A Lee

**Affiliations:** Computer Systems Laboratory, Department of Computer Engineering, Chosun University, Gwangju Dongku SeoSuk Dong 375, Gwangju City 501-759, Korea; E-Mail: saniabba2004@gmail.com

**Keywords:** wireless sensor networks, autonomous self-awareness and adaptive, routing technique, fault tolerant, route repair, self-healing, ASAART

## Abstract

We propose an autonomous self-aware and adaptive fault-tolerant routing technique (ASAART) for wireless sensor networks. We address the limitations of self-healing routing (SHR) and self-selective routing (SSR) techniques for routing sensor data. We also examine the integration of autonomic self-aware and adaptive fault detection and resiliency techniques for route formation and route repair to provide resilience to errors and failures. We achieved this by using a combined continuous and slotted prioritized transmission back-off delay to obtain local and global network state information, as well as multiple random functions for attaining faster routing convergence and reliable route repair despite transient and permanent node failure rates and efficient adaptation to instantaneous network topology changes. The results of simulations based on a comparison of the ASAART with the SHR and SSR protocols for five different simulated scenarios in the presence of transient and permanent node failure rates exhibit a greater resiliency to errors and failure and better routing performance in terms of the number of successfully delivered network packets, end-to-end delay, delivered MAC layer packets, packet error rate, as well as efficient energy conservation in a highly congested, faulty, and scalable sensor network.

## 1. Introduction

Autonomous self-aware and adaptive systems provide the means to solve the computational complexities of a dynamic environment. Autonomous self-aware routing algorithms possess the capability of configuring, healing, optimizing, and protecting themselves autonomously [1,2,3,4,5,6,7,8,9,10,11,12,13,14,15,16,17,18,19,20]. This also entails the ability to find the best means for optimizing performance in resource-constrained environments. Sensor networks are exposed to inhospitable environments and need to be adapted to changes in the environmental parameters or users. For example, a sensor network deployed to track and trace enemy positions in a battlefield must adapt to changes in the enemy position. In addition, a sensor network that helps the robot in a car manufacturing factory must provide the robot with extended situational awareness in its vicinity to adapt to the movement of the robot for better functioning of the system. Sensor networks that are resource constrained have low power, low communication, and minimal computational capabilities. In addition, they possess low storage capabilities, low cost, reliability and fault tolerance, which are the critical sensor network design considerations [1,2,3].

Sensor nodes may fail for various reasons. These include radio interference, battery failure, and synchronous and asynchronous communication mechanisms. These failures mainly arise as a result of software or hardware faults, malicious code, environmental changes, and timing closure. On the whole, the result of such an event is that a sensor node becomes inaccessible or disrupts certain operating conditions that are critical to providing the required service. Communication in sensor networks is highly critical because the links in a network are prone to faults and errors. For instance, the collision of network packets may result in a failure. The presence of such a failure in a routing protocol will lead to considerable network packet drops resulting in excessive network delays and consumption of a substantial amount of energy [10,11,12,13,14,15,16,17,18,19,20,21,22,23,24,25,26,27,28,29,30,31,32,33,34,35,36,37,38,39,40,41,42,43,44,45,46,47,48,49,50].

This research was motivated mainly by the observation that the traditional fault-tolerant approach to routing in a wireless sensor network introduces more redundant transmission and retransmission of network packets and consumes an excessive amount of energy. In addition, they are also deficient in terms of their autonomous self-awareness and adaptive capabilities in routing packets from their sources to their destinations in the presence of transient and permanent node failures and in highly scalable and congested networks. The essential concerns here are how to avoid the problem of packet flooding because of broadcasting and network scalability issues. To address these issues, our approach attempts to reduce the number of redundant network packets caused by flooding. Both the SSR [40,41,51,52] and SHR [24,53] routing protocols make use of a prioritized back-off delay for forwarding packets, with only the receiver of the forwarding packets reaching the destination. Our approach focuses on the minimization of these control packet overheads and efficient end-to-end data delivery. In doing so, we combined both the continuous and slotted prioritized transmission back-off delays to obtain local and global network state information and a multiple randomize function for faster routing convergence and efficient and reliable route repair in the presence of transient and permanent node failures. In our proposed scheme, the sensor node that transmits the packets contains information pertaining to its neighbors in the control header packets. The sender node is self-aware and adaptive if it knows both local and global state information contained in the control packets. In this case, the receiver node knows whether its neighbors have been covered based on the global information obtained in the neighbors’ control packet list information. The sender node with the auto-self-awareness back-off information now has a higher priority to forward the network packet to the destination. This approach will provide resiliency to errors and failures, minimize the end-to-end network delay, and improve the overall network routing performance and energy efficiency.

Quite a few number of approaches to adaptive and fault tolerance in sensor networks are given in the literature [12,13,14,15,16,17,18,19,20,21,22,23,24,25,32,33,34,35,36,37,38,40,41,50,51,52,53]. However, most proposed approaches do not provide quantitative techniques for autonomic self-aware and adaptive fault tolerant routing features in a wireless sensor network.

Common approaches to multi-hop routing employ routing tables that show the path from sources to destinations. The most popular of these are the Dynamic Source Routing (DSR) [54], *Ad-hoc* On Demand Distance Vector (AODV) [55], MintRoute [25], Low Energy Adaptive Clustering Hierarchy (LEACH) [5] and Directed Diffusion (DD) [56]. These approaches consider the state information of the sensor node neighbor as active or sleeping in order to make routing decisions. These routing schemes incur more overhead if autonomic self-aware and adaptive features are not integrated. Therefore, providing an autonomic self-awareness and adaptive mechanism to fault tolerant routing is a better way for addressing the challenges of inhospitable and hostile environments.

Self-Selective Routing (SSR) [40,41,51,52] and Self-Healing Routing (SHR) [24,53] are other approaches for providing fault tolerance and recovery techniques to sensor networks. In SHR, a sensor node broadcasts its packets to all its neighbors within its communication range. Based on hop distance knowledge and a priority schedule coupled with a back-off delay scheme, the neighbors ensure that the network packet is transmitted by only one of the contending neighbors. However, the lack of an autonomic self-awareness and adaptive feature makes these two proposed fault tolerant routing algorithms prone to errors, failures, and associated routing overhead. In this research paper, we present an autonomous self-aware and adaptive fault tolerant routing technique that addresses the limitations of self-healing and self-selective routing schemes for reduced packet contention, route repair, and efficient energy conservation in the presence of transient and permanent node failure rates and in a highly congested and scalable sensor networks. The salient contributions of this research paper are as follows:

1. We propose an autonomous self-aware and adaptive fault tolerant routing technique for efficient and reliable route formation and faster routing decision-making for wireless sensor networks. This is achieved by combining both continuous and prioritized slotted back-off delay and multiple randomize function techniques for speedy routing convergence to obtain local and global network state information for the efficient and reliable routing of sensor data.
2. We propose a route repair technique using the greedy algorithmic approach for reliable transmission of sensor data in the presence of permanent and transient node failure rates, and efficient adaptation to simultaneous network topology changes.
3. We conducted an extensive simulation to demonstrate the routing performance, flexibility and efficiency of the autonomous self-aware and adaptive fault tolerant routing technique (ASAART) under five different simulated scenarios. The simulation results show that the ASAART performs better in terms of high resiliency against errors and failure, and has better routing performance and energy efficiency compared with SSR and SHR protocols in the presence of transient and permanent node failure rates and in highly congested, faulty, and scalable sensor networks.

The rest of the paper is organized as follows: Section 2 presents related literature; Section 3 discusses the approach for autonomous self-awareness and adaptation to routing in wireless sensor networks; Section 4 discusses the SSR and SHR protocols; Section 5 discusses our proposed routing technique and route repair mechanism; Section 6 discusses the performance evaluation and simulation results; and Section 7 provides concluding remarks and plans for future enhancements.

## 2. Related Work

Quite a few number of related works have been conducted with adaptive and fault tolerant routing algorithms for wireless sensor networks [12,13,14,15,16,17,18,19,20,21,22,23,24,25,32,33,34,35,36,37,38,40,41,50,51,52,53]. For instance, Gilbertt *et al.* [40,52] proposed a self-selective fault tolerant routing protocol for a wireless *ad hoc* network. The protocol employs a combined radio broadcast and autonomous programming technique to implement routing with reduced overhead. The routing protocol routes the packets via a near optimal path between nodes at runtime. Simulation results showed high routing performance under heavy network traffic, as well as network node and link failures. The authors in [24,53] presented a novel protocol for self-healing in sensor network routing. The approach employs broadcast transmission and prioritized slotted back-off delay for sensor nodes to use their hop distance from the destination in order to determine how to transmit packets. By doing so, this ensures a dynamic traversal of minimum routes without nodes that specifies those neighboring nodes that transmit network packets. However, when faulty routes are encountered, the scheme locally and dynamically re-routes packets in order to survive the shortest routes despite spontaneous network topology changes.

Other approaches that avoid neighbor state maintenance and allow destination nodes to contend with transmitting network packets are given in [26,27]. These two protocols are similar to SHR [24,53] and SSR [51,52], which use local information instead of global network state information. Unfortunately, none of the two proposed approaches provide route repair mechanisms. GRAB [26] employs a very complex fault tolerant mechanism by allowing the flow of redundant network packets to take multiple paths to destinations. Hellsenbttel *et al.* [28] employed a location coordinate system to allow only destination nodes within a given region to communicate with each other. Zori and Rao [29] used a concept similar to [28], and incorporated a back-off delay mechanism. Such mechanism makes use of a dual-radio concept with a busy tone signaling to enforce the channel to be clear before transmitting data in order to minimize the probability of network packet collisions. Another approach is given by Blum *et al.* [30], who employed an eligibility region given as a 60% fan shape that extends from a source towards a destination. Upon this, if a source node does not “hear” a response from any of its neighbors, it moves the eligibility region and looks for other neighbors. All these approaches use packet forwarding techniques, *i.e.*, back-off delay and RTC/CTS schemes that result in packets that forward overhead.

Other methods for direct routing are given in DSR [54], AODV [55], and DSDV [31]. The work presented by Chokers and Elizabeth [55] is regarded as the most popular directed routing protocol. Such protocol uses the route request and a reply mechanism in order to set up network paths between sources and destinations. This process yields a routing table by each node that contains a path from the sources to destinations. Johnson *et al.* [54] employ a technique where the source node should contain the complete route information in the packet header. This also means that multiple routes are stored at the source node in order to avoid node failure. The limitation of the DSR protocol is its increases in the amount of memory use of the source node because of intermediate node storage and routing overhead. AODV [55] employed the use of a hello message in order to detect and fix broken network links. This results in an increase in network latency, particularly when dynamically looking for new routes.

Gelenbe and Liu [51] presented a cognitive and quality of service approach to routing in wireless sensor networks. They used the concept of smart packets for network path discovery coupled with reinforcement learning and neural networks. In addition, they used the ant colony concept to mimic the pheromone-based technique ants use to find a given path and communicate the information to colony members. Experiment results showed the efficiency of their approach in adapting to network changes over time. Self-adaptive routing in multi-hop sensor network was presented by Bourndenas *et al.* [32]. A study of an effective method that uses time series analysis to forecast the occurrence of errors and faults was presented. The work considered an autonomic routing service through adaptation to avoid areas where failure or errors are expected. Simulation results showed the benefit of their approach.

The authors in [38,39] compared the performance of three routing protocols, namely, the Multi-Parent Hierarchical (MPH), AODV, and DSR protocols. The protocols are evaluated both when exposed to different “jamming” attacks and without attacks, and considering diverse locations of the jammer. Simulation results show that MPH routing has greater immunity for tolerating attacks than DSR and AODV. This is because MPH reduces and encapsulates the network segment when subjected to a network attack. In addition, the self-configuring features of MPH yield higher resiliency and better routing performance compared with AODV and DSR protocols. Del-Valle-Soto *et al.* [39] proposed metrics for efficient energy consumption in wireless sensor networks. They compared the performance of three routing protocols, namely MPH, AODV, DSR, and Zigbee Tree Routing (ZTR). Simulation results demonstrated the impacts of communication metrics on performance, throughput, reliability, and energy consumption. They showed that MPH achieved 19.3% reduction of network packet retransmissions, 26.9% reduction in routing overhead, and 41.2% increase in protocol recovery from topology failure compared with AODV routing. In addition, their approach achieved 15.9% decrease in energy consumption compared with AODV, 13.7% compared with DSR, and 5% compared with ZTR protocols. Apart from the few related works mentioned above and in the literature in this paper, we propose an autonomous self-aware and adaptive fault-tolerant routing technique for wireless sensor networks. We integrate the autonomic self-aware and adaptive mechanism for route formation and repair in the presence of transient and permanent node failure rates in order to achieve high routing performance, energy efficiency and resiliency against errors and failure of sensor nodes, and adapt to simultaneous network topology changes.

## 3. Autonomous System

An autonomous system is one which identifies its operating environment and senses the operating parameters, transforms its behavior in that environment, and adapts to the situational changes of the environment [6,7,8,9,10,11,13,14,15,16,17,18,19,20]. Autonomic systems provide a means to address the system’s complexities by employing technology to manage and control complex and dynamic systems. Such systems work with independent and predefined conditions, policies, rules *etc.* without human involvement. They can configure, optimize, manage, and control their actions based on predetermined conditions, rules, and acquired knowledge of the operating environment for a given period. The word autonomic originates from the field of human biology. For instance, the autonomic nervous system in the human body monitors and controls our heartbeat, checks blood sugar levels, and regulates and maintains body temperature normal without human intervention. An autonomous self-aware system can be employed in wireless sensor network to solve routing of sensor data without human intervention. In autonomic computing, self-managing capabilities in the system are achieved by employing suitable actions according to situational changes that arise in a dynamic environment. The main goal of autonomic systems is to control the loop that sources and gathers detailed information from the environment, and then take appropriate actions. According to Kephart and Chess [6], there are four characteristics of an autonomic system, as follows:

**Self-Configuration:** In wireless sensor networks, self-configuration is the ability of sensor nodes to adapt dynamically to environmental and situational changes based on the predefined stated policies and rules that govern the operating environment. This entails the deployment of new sensors, removal of faulty components, or unpredicted situational changes that may arise in a dynamic environment. The use of dynamic adaptation provides continuous strength in sensing and enhances sensor node productivity, which gives rise to the scalability and flexibility of sensor networks.

**Self-Healing:** This is one of the characteristic features of autonomic systems that provide the means for autonomic route discovery, fault detection, and recovery in wireless sensor networks. Such feature is the ability to discover, diagnose, and react to unpredicted situations that may arise in a dynamic environment. Self-healing sensor network components and routing techniques can detect network malfunction and initiate a route repair technique without affecting the entire sensor network. The route repair technique makes the network resilient against errors and faults.

**Self-Optimization:** This attribute provides autonomic monitoring and control to facilitate fair and optimal use of available resources. Self-optimizing sensor components have the ability of controlling themselves to achieve the goals and policies stated in a given dynamic environment. Such control measures are the reallocation and rescheduling of available resources caused by arising situations in the dynamic environment in order to enhance overall network utilization and real-time transmission of sensory information.

**Self-protection:** This characteristic of autonomic systems provides sensor networks with the capability of anticipating, detecting, identifying, and providing protection mechanisms against threats and other variant attacks. Self-protecting sensor components can detect malicious and suspicious behavior within the network, and take reactive measures to counterattack actions in order to make the network less vulnerable to threats. Self-protecting features of sensor network components provide security measures and policies to protect components and the entire network against any internal or external threats and vulnerability attacks. In conclusion, when sensor network components possess these four characteristics, the network can configure, heal, optimize, and protect its operation, and enhance routing performance [6,7,8,9,10,11].

### 3.1. Approach to Autonomous Self-awareness and Adaptation for Routing in Wireless Sensor Networks

Autonomous self-aware and adaptive systems are a proven solution for overcoming the complexity imposed by sensor network routing algorithms [1,2,3,4,5,6,7,8,9,10,11,12,13,14,15,16,17,18,19,20,21,22,23,24,25,26,27,28,29,30,31,32,33,34,35,36,37,38,39,40,41,42,43,44,45,46,47,48,49] and so are self-healing systems [6,7,8,9,10,11,12,13,14,15,16,17,18,19,20,21,22,23,24,51,52,53]. Self-aware and adaptive systems are better methods for enhancing and improving the potential for understanding environmental situations and complex dynamic environments. In order to have a better understanding of the overall systems’ capability and awareness to acquire and process information, five levels of awareness need to be considered. Such levels describe the extent and worthiness to which a system can adapt, and the extent that the respective adaptation effects have on the system [7,8,9,10,11,12]. In this paper, we consider the five levels of self-awareness given in [9], which are:

**Event Awareness:** This is the main level of self-awareness. This level focuses on the self-description of sensor network entities and how the events associated with these entities relate to other entities in the network system. It involves using basic information processing for sharing among network entities in the entire network. For dynamic adaptation, the self-aware routing technique must not establish a local routing decision only, but consider both local and global state information, and a defined boundary between self-awareness and adaptation. This boundary has to be considered at run-time based on the routing function and tuned parameters [13,14,15,16,17,18,19,20].

**Situation Awareness:** At this level of awareness, several techniques and principles are joined to form a consistent reasoning both at the local and global level situational awareness. At this level, the routing function is advanced to specify a property between the sensor’s network entities that are nodes and links. Several methods and techniques can be employed to ensure reliable and efficient understanding of the situational system. For example, fuzzy logic and probabilistic models can be used at this level [33,34,35,36,37,38,39,40,41,42,43,44,45,46,51]. Fuzzy models can be employed to describe the probability of error or failure occurring in the routing function. The routing function requires further information about the network to detect and characterize higher-level situations. The most important aspect of this level is the information abstraction at a higher level and efficient dissemination of information sharing between sensor nodes in the network [18].

**Adaptability Awareness:** This level of awareness provides support for network entities to detect the adaptability of other network components and provide their own adaptation interface. By doing so, the sensor nodes can select the best neighbors to meet the self-adaptation goal, *i.e.*, find the best routing path. At this level of awareness, different methods and techniques can be employed to ensure that the routing algorithm possesses the capability of adapting to situational changes.

**Goal Awareness:** This level of awareness defines the goals or the objective function to be achieved by the routing algorithm. This involves combining several different actions, goals, adaptations, and conflicting goals for the efficient routing of packets in the network, and for resource utilization. The routing function needs to specify an objective function that can be minimized or maximized at runtime. Goal awareness entails runtime goal checking to determine dynamically those goals that can be satisfied for a given constraint resource and threshold limit [6,18].

**Future Awareness:** This level of awareness is concerned with a thorough knowledge of the entire sensor network state. These include network cost, energy consumption, overhead, reliability, *etc.* This information helps reduce the number of actions to be taken in the future. Similarly, monitoring the network state can lead to underlying data for future resource volume and utilization. Furthermore, the knowledge gained for the correlation of resource lifecycles with the activity patterns detected at the situation awareness level can inform the possible actions and conditions that lead to poor resource routing and information unavailability. Therefore, at this level, adaptive identification of the network state information is the major goal associated with this level [9,10,11,12,18]. By way of integrating these awareness levels into SHR, the problem associated with the routing of sensor data in wireless sensor networks can be solved.

### 3.2. Modular Approach to Fault Propagation in Wireless Sensor Networks

In order to integrate autonomous self-aware and adaptive mechanisms into fault tolerant routing for sensor networks, it is essential to explain the basic difference between faults, errors, and failure [10,11,12]. In sensor networks, a fault is any type of sensor node defect that leads to error, for instance, a loose sensor node connection in the network. An error is an undefined state of the node condition such that the condition or state leads to a node failure, *i.e.*, the state of the system when transmitting and retransmitting sensor data. In a sensor network, sensor node failure implies the manifestation of error. This occurs when a sensor node cannot perform its functions and violates the network design specification, which happens when sensor nodes cannot transmit their data within a specified time interval. To integrate self-awareness functionalities into sensor networks in order to provide resiliency against faulty conditions, fault detection and recovery measures are required. In fault detection, some design techniques need to be incorporated into the routing algorithm in order to detect that a specific functionality of the sensor nodes is or will be faulty. On the other hand, after a fault is detected, the routing algorithm should be able to prevent or recover from it. This ensures smooth and steady packet routing from sources to destinations [13,14,15,16,17,18,19,20,21,22,23,24,25,26,27,28,29,30,31,32,33,34,35,36,37,38,39,40,41,42,43,44,45,46,47,48,49].

**Figure 1:**
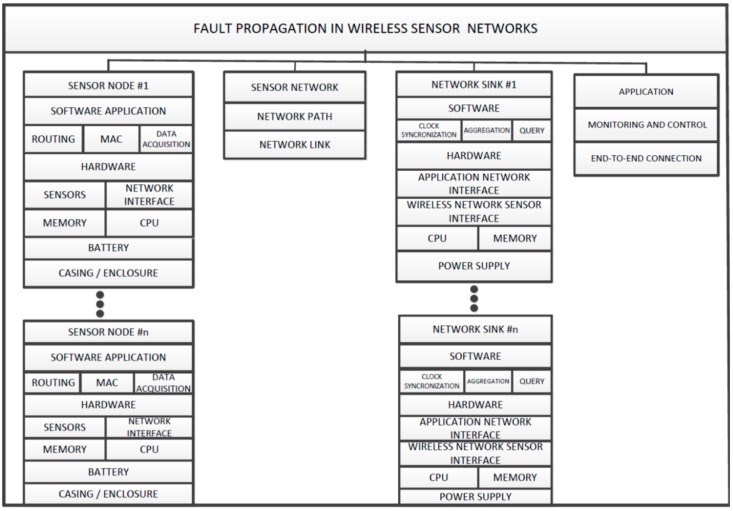
Modular approach to fault propagation in wireless sensor network.

The emergence of wireless sensor networks presents a challenging issue because of its deployment in harsh and inhospitable dynamic environments. Sensor nodes are subjected to a fault that occurs in many components of the sensor network. In order to illustrate how a fault is propagated in different sections of the sensor network system, we propose a modular architecture in contrast to the layered architecture presented in [12]. As illustrated in Figure 1, a fault in each module cannot be propagated to other modules in the system. For instance, when the node battery power is down, this causes only the node to be down without affecting other modules. In layered approach, the failed node affects the operation of other nodes. If this node is in the critical path of the network, the packets of other nodes that depend on the critical path will not reach their destination until the path is recovered or restored. In addition, if the application module components, *i.e.*, data, and users suffer a faulty software bug or hardware failure for the sensor node, the overall system is considered to be faulty as well. With the modular approach to fault propagation in sensor networks, fault occurrences are limited within their module without affecting other module functionalities. Below are the fault explanations in different modules of the sensor network:

**Sensor network node faults:** The sensor node module is comprised of several hardware and software components that can cause the system to malfunction. For example, the battery power can go down, which can cause the system to fail. Similarly, the casing or system enclosure can suffer from environmental effects, thereby exposing the hardware components of the sensor node to harsh environments. This can cause problems such as short-circuiting, and then sensor readings would be incorrect. Furthermore, the data acquisition readings may be subjected to errors if the underlying sensors provide incorrect sensor measurements. Another important issue that software bugs can also cause errors in wireless sensor networks [10,11,12,13,14,15,16,17,18,19,20,21].

**Sensor network faults:** Network faults are the result of routing, which is the most essential building block of sensor networks. The presence of faults in the routing function can result in massive network packet drops or too much network delay [10,11,12]. Communication in wireless sensor networks is highly critical because links are highly volatile to faults and errors. One source of link failure in wireless sensor networks is the collision of network packets. For instance, the authors in [23,43,44,45,46] studied the potential for network packet collisions in a nearby network caused by phase change and overlap. On the other hand, sensor nodes might have a perfect link connection, but the packets might not be transmitted to their correction destination because of path errors. Finally, a software bug in the routing function can create deadlocks or transmit the packets to the wrong destination [10,11,12].

**Network sink faults:** The function of the network sink is to collect all sensor data generated in the network and transmit them to the application module. This part of the wireless sensor network can also be subjected to faults and errors from its components. Sink node failure would not render the collected data useless, unless backup or redundant sinks were present. With a modular approach, sink node failure should not affect the functioning of the entire network, as illustrated in Figure 1.

## 4. Self-Selective Routing (SSR)

In this section, we provide a brief explanation on SSR [40,41,51,52] and SHR [24,53]. In SSR, the network nodes know their distance through the mechanism of route request and reply. In this scheme, the nodes broadcast packets with an estimated distance to the destination neighbors. The node then employs the SSR protocol to determine autonomously the node that should transmit a given packet to the next node using a prioritized transmission backup delay. Upon receiving the packet, the node then schedules further packet transmission immediately with a random delay that is proportional to its path length from the destination. The transmission back-off delay is expressed using the mathematical expression given in Equation (1) as [40,41,51,52]:

(1) $${Trans\_Back\_off\_}_{Delay}=\{\begin{array}{c} \beta *\left(\alpha -\;\alpha_{expected}*\text{Random}\left(0,1\right)\right)\;\text{if}\;\text{α}\;>\;\alpha_{expected}\\ \frac{\beta }{\alpha_{expected}-\;\alpha +1}*\text{Random}\;\left(0,1\right)\;\text{if}\;\text{α}\;\leq \;\alpha_{expected} \end{array}$$

where α is the hop count of the destination node, and $\alpha_{expected}$ is the sender node expected hop counts in the destination reply packets. The random function is the random number generator that produces real values uniformly distributed between the interval [0,1]. This random function is used to randomize the delays and reduce packet collisions. The parameter β is a scaling factor used to stretch the random delay values generated by the randomize function. Equation (1) assigns a *Trans_Back-off__Delay_* greater than β to those nodes with hop counts that are greater than α_expected_. In this case, the smaller the value of α, the least is the Trans_Back_off__Delay_, and the node has the highest probability of transmitting the packet [40,41,51,52]. The limitations of SSR are:

1. The SSR routine computes a node’s transmission back-off delay in continuous time, in contrast to slotted time. The disadvantage is that it has a higher rate of packet collisions.
2. Because of non-zero probability, the packets may travel a longer route while a shorter route exists that increases network delay.
3. In SSR, there is no route repair mechanism for propagating packets around faulty routes.

### 4.1. Self-Healing Routing (SHR)

SHR [24,53] employs the concept of a prioritized time-slotted transmission back-off delay and route repair routine. The first improvement on SHR is as follows: when a given node receives a packet, the node employs Equation (2) instead of Equation (1) to compute the delay before transmitting the packet:

(2) $${Trans\_Back\_off\_}_{Delay}=\{\begin{array}{c} \beta *\left(\alpha -\;\alpha_{expected}+Random\;\left(0,1\right)\right)\;if\;\alpha \;>\;\alpha_{expected}\\ \frac{\beta }{\alpha_{expected}-\;\alpha +1}*Random\left(0,1\right)\;if\;\alpha \;\leq \;\alpha_{expected} \end{array}$$

From Equation (2), we can determine that the computed delay is such that the nodes closest to the destination forward their packets, rather than those faraway. Furthermore, Equation (2) assumes that there are delays, and thus ensures the response of those nodes far away from the destination than the sender, according to their distance from the destination. In doing so, no packets travel further than the necessary routes, even if there are no nodes closer to the destination: Another addition to SHR is the transmission back-off delay in the slotted time, in contrast to the continuous time used in SSR [40,41,51,52]. The transmission back-off delay is given by Equation (3) as [24,53]:

(3) $${Trans\_Back\_off\_Delay}_{SLOTTED}=\lfloor \frac{{Trans\_Back\_off\_}_{Delay}}{Wds}\rfloor *Wds$$

where Trans_Back_off__Delay_ is as given in Equation (2), and *Wds* is the width of the used slot.

### 4.2. Route Repair in Self-Healing Routing (SHR)

Another fundamental addition to SHR [24,53] is the inclusion of a route repair mechanism for propagating packets across faulty routes. According to SHR, a faulty route is that where a source node is the one with the minimum distance to the destination compared with its neighbors. In this situation, based on the topology condition, a suitable method needs to be considered to re-route the packets across the shortest route. In SHR, this is achieved by simply adjusting the hop counts of the sending node. Below are the steps for implementing route repair in SHR using the DATA and HELLO packets.

For sending packets:

1. From the beginning, before transmitting an unseen packet, a resend bit is set in the packet header to zero, and then the timer timeout is set; subsequently, the packet is transmitted to the destination.
2. In case there is no response apart from the previous sender acknowledgement (ACK) before the timer expires, the packet is resent by setting the reset bit to one.
3. Else, if a response is received, an explicit ACK packet is sent. However, if the expected hop distance of the respondent is not one less than the hop count, the hop counts that correspond to the packet destination are substituted with one greater than the expected distance.
4. If there is no response to a retransmitted packet, a higher protocol layer is signaled to take the next action. In this case, the source or sender now becomes isolated, and there is no need for transmission.
5. After receiving ACK for a retransmitted packet, the hop count of the packet destination is increased by two, and the maximum of the current and expected hop count from the response packet. Next, an explicit ACK is sent [24,53].

For packet receivers:

1. For a given network packet with a resend bit initially set to zero, if the packet destined for a particular destination has already been received, do nothing.
2. Otherwise, continue as normal and compute the transmission back-off delay using Equations (2) and (3).

In our approach, we address the limitations of the SHR and SSR routing protocols by using combined continuous and slotted prioritized transmission back-off delay to obtain local and global network state information and multiple randomize function for faster routing convergence and efficient and reliable route repair technique in the presence of transient and permanent node failure rates.

## 5. Proposed Approach to Autonomous Self-awareness and Adaptive Fault Tolerant Routing Technique (ASAART)

In order to demonstrate our approach using autonomous self-awareness and adaptive routing techniques, namely ASAART, we consider low duty-cycle wireless sensor networks [14,15,16,17,18,19,33]. In this type of network, the sensor nodes remain sleeping most of the time and wake up asynchronously in the case of event transmission. Therefore, the concept of probabilities that forwards a decision based on the delay distribution of the destination nodes can be employed [1,2,3,4,5,6,7,8,9,10,11,12,13,14,15,16,17,18,19,20,21,22,23,24,25,26,27,28,29,30,31,32,33,34,35,36,37,38,39,40,41,42,43,44,45,46,47,48,49,50,51,52,53,54,55,56,57]. In doing so, only packets are transmitted in order to achieve shorter delays and reduce packet redundancy. The probabilistic approach signifies that when a sensor node receives a network packet, the packet is forwarded with given probability δ. Such probability δ is computed by the network state information obtained from individual neighboring nodes. The routing decision is computed using the improved formula given in Equations (4)–(10). The SHR [24,53] and SSR [40,41,51,52] algorithms use more redundant transmission and retransmission of packets. Our approach attempts to reduce such redundant packets. SHR and SSR make use of prioritized back-off delay for forwarding packets, and only the receivers of the forwarding packet reach the destination. Our approach focuses on the reduction of these control packet overheads and efficient data delivery ratio. Rather than using local information to route a given packet to the desired destinations, our approach combines both the local and global state information in order to avoid faults and collisions in the network.

The most important issues to address are: (1) at the initial phase of network construction, how to avoid the problem of packet flooding because of broadcast and (2), when the network is scaled to a large network, the broadcast packet will overflow the network, thus causing too much delay and packet collisions that consume much energy. In order to address these issues, the autonomous self-awareness and adaptive scheme proposed in this paper will ensure reliable and efficient transmission and retransmission of network packets. For the self-awareness scheme, the sensor node that transmits the packets contains information pertaining to its neighbors in the control packet. The node is self-aware and adaptive if it knows all the local and global information on the packet. Then, the receiver node knows whether its neighbors have been covered based on the global information obtained in the neighbor’s control packet list information. In this case, the sensor node will compute the auto self-awareness back-off delay according to Equations (4)–(10) in order to obtain the entire network state information. The sensor node with the auto self-awareness back-off information now has higher priority to forward the network packet. In this case, the sensor node that uses the global information of its neighbors has a very low back-off delay. The self-awareness and adaptive features of the node’s neighbor network state information minimizes the network end-to-end delay and enhances the entire network routing performance. Here, we define global, local, and auto-self-awareness back-off delays as given in Equations (4)–(10). Ideally, this combines both the local and global network state information to have full autonomic self-awareness of the entire network state information, and reduces the time to converge to minimal routing path for efficient packet routing throughout the entire network [24,40,41,42,43,44,45,46,47,48,49,51,52,53].

Herein, we define the parameters used in Equations (4)–(10). ${Hopcount}_{Dest}$ is the number of hop counts from the current node to the destination; ${NumHopCount}_{expected}$ is the expected number of hops contained in the destination reference packet header information; ${Scale}_{Factor}$ is a parameter used to stretch the random function in order to avoid network packet collision; Rand (seed) is a random function generator that produces random floating point numbers within the interval [0,1] based on the value of the seed; and Slot_size is the size of the slot or the transmission window width. The global awareness transmission back-off delay is computed for each node using Equations (4) and (5):

If ${Hopcount}_{Dest}\;>\;{NumHopCount}_{expected}$

${\mathrm{Global-Awareness-Back-off}}_{\mathrm{Delay}}=$

(4) $${Scale}_{Factor}*\left({Hopcount}_{Dest}-{NumHopCount}_{expected}*Rand\left(0,1\right)\right)$$

*Else* ${Hopcount}_{Dest}\;\leq \;{NumHopCount}_{expected}$

(5) $${\mathrm{Global-Awareness-Back-off}}_{\mathrm{Delay}}=\frac{{\mathrm{Scale}}_{\mathrm{Factor}}}{{\mathrm{NumHopCount}}_{\mathrm{expected}}-\;{\mathrm{Hopcount}}_{\mathrm{Dest}}+\;1}*Rand\left(0,1\right)$$

The local awareness transmission back-off delay is computed for each node using Equations (6) and (7), depending on whether the hop counts from the destination parameter is greater, less than, or equal to the sender’s node number of expected hop counts:

If ${Hopcount}_{Dest}\;>\;{NumHopCount}_{expected}$

${\mathrm{Local-Awareness-Back-off}}_{\mathrm{Delay}}=$

(6) $${\mathrm{Scale}}_{\mathrm{Factor}}*\;({\mathrm{Hopcount}}_{\mathrm{Dest}}-{\mathrm{NumHopCount}}_{\mathrm{expected}}+Rand\left(0,1\right))$$

*Else* ${Hopcount}_{Dest}\;\leq \;{NumHopCount}_{expected}$

(7) $${\mathrm{Local-Awareness-Back-off}}_{\mathrm{Delay}}=\;{\mathrm{Global-Awareness-Back-off}}_{\mathrm{Delay}}$$

The autonomous self-awareness technique combines both the local and global back-off delay transmission strategies in order to find the entire network state information for efficient transmission and retransmissions of packets. Equation (8) combines two randomized functions to allow both the near and far nodes minimize the back-off delay. Based on this, the sender node has a greater probability of transmitting the packet:

If ${Hopcount}_{Dest}\;>\;{NumHopCount}_{expected}$

(8) $$\begin{array}{l} AutoSelf-Awareness-Back-off_{Delay}=\\ Scale_{Factor}*\;(Hopcount_{Dest}-NumHopCount_{expected}*Rand\left(0,1\right)+\;Rand\left(0,1\right)) \end{array}$$

*Else* ${Hopcount}_{Dest}\;\leq \;{NumHopCount}_{expected}$

(9) $$AutoSelf-Awareness-Back-off_{Delay}=\;Global-Awareness-Back-off_{Delay}$$

From Equation (8), it is clear that the auto self-awareness back-off delay assigns a delay greater than *Hopcount_Dest_* to nodes with a hop count greater than *NumHopCount_expected_* parameters. Therefore, the smaller the value of the *Hopcount_Dest_* parameter, the smaller is the *AutoSelf-Awareness-Back-off_Delay_* parameter and the node has the highest probability of transmitting the packet. The slotted autonomous self-aware back-off slotted formula is as expressed in Equation (10) as [24,40,41,51,52,53]:

(10) $$\begin{array}{l} AutoSelf-Awareness-Back-off_{Delay\_Slotted}=\\ \;\;\;\;\lfloor \frac{\text{AutoSelf}-\text{Awareness}-\text{Back}-\text{offDelay}}{Slot\_Size}\rfloor *Slot\_size \end{array}$$

Where the parameters AutoSelf−Awareness−Back−offDelay are given in Equations (8) and (9), and Slot_size is the size of the slot window used. Equation (10) provides the autonomous self-aware and adaptive back-off delay that combines both the continuous and slotted time interval. This approach lowers the back-off delay, and the sensor nodes have the greatest probability of transmitting the packets. In addition, the approach provides the current node with both local and global network state information in order to reduce end-to-end delay for efficient and reliable packet transmission.

### 5.1. Autonomous Self-Aware and Adaptive Route Repair Technique in ASAART

**Figure 2:**
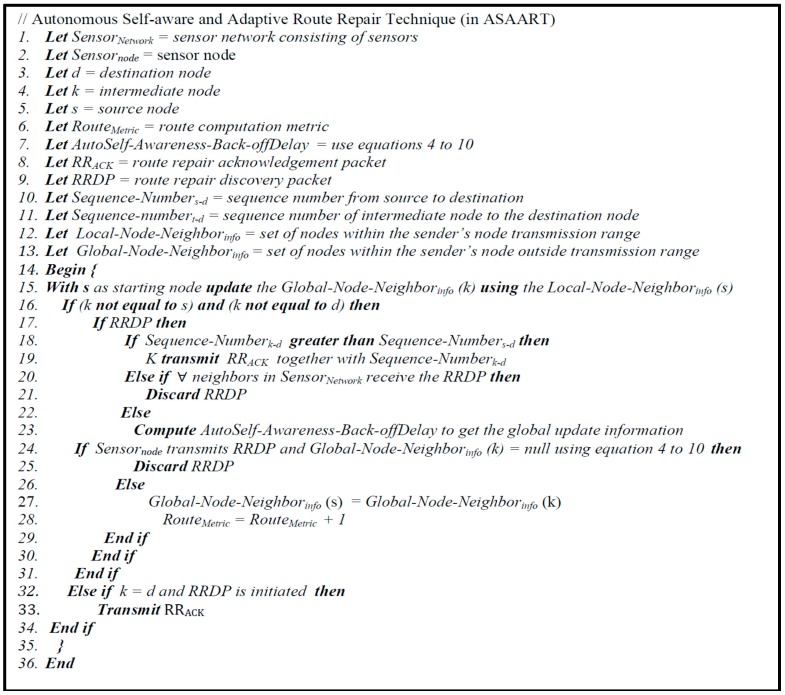
Proposed autonomous self-aware and adaptive route repair technique in (ASAART).

Herein, we employ the greedy algorithm [33,58] to implement the autonomous self-awareness and adaptive route repair technique. By implementing the concept of the self-awareness principle defined in Section 3, the sensor nodes become self-aware and adaptive for both local and global network state information. During route maintenance, the source node initiates a new route repair packet (*RRP*). The route repair packet *RRP* consists of the source node identification (*Source_id_*) number, packet identification number (*packet_id_*), route metric (Route_Metric_), back-off delay (*AutoSelf-Awareness-Back-off_Delay_*), and route metric sequence number of sources to destination (*RouteMetric_Seqn_*). In this case, when a sensor node *k* receives an *RRP* packet, it processes the packet according to the algorithm given in Figure 2. 

There are two sets of nodes, one that consists of the node’s neighbors within its transmission range (*Local-Node-Neighbor_info_*), and another that is outside the transmission range that leads to the minimal routing path (*Global-Node-Neighbor_info_*). At the initial stage of network creation, sensor node *k* updates its global state information *Global-Node-Neighbor_info_* (*k*) using the source node’s local neighborhood information (*Local-Node-Neighbor_info_*) to update its global node information. This approach helps to prevent and avoid both transient and permanent faults and failure in the network. A detail of the route repair technique in ASAART is illustrated in Figure 2.

### 5.2. Reliable and Secure Route Formation in ASAART

In the ASAART routing protocol, the route discovery packets are transmitted by the source node through the packet broadcast mechanism [32,33,34,35,36,37,38,39,40,41,42,43,44,45,46,47,48,49]. In this case, there is a need to control the continuous flooding of the route discovery packets, particularly in a very dense network. In our scheme, shown in Figure 2, ASAART uses the sender node local information that is contained in the control packet. Upon receiving the packets from the sender node, it uses the local neighbor information to update its global network state information contained in the control packet header information. The node then computes the auto self-awareness back-off delay based on the expected number of hops to the destination, as given by Equations (4)–(10). During the computation of the auto-self-awareness back-off delay, the sender node listens to the channel and prevents the transmission of the data packets. This happens when the sender node overhears that one of its neighboring nodes with the expected hop count to the destination has already transmitted the data packets. However, if the sender node does not overhear its neighbor’s transmission and its back-off timer fires, the sender node will now update its expected hop count in the data packets and then re-transmit the data packets to the destination. The sender node with the auto self-awareness back-off information will have the highest priority to forward the packets to the next intermediate or destination node. To reduce the problem of redundant transmission, after the destination node receives a route discovery packet, it sends a route discovery acknowledgement to the source node. ASAART uses an efficient flooding control scheme that minimizes the redundant transmission of network packets. In this case, the intermediate and destination nodes have the complete network state information for a reliable route formation based on the discovery packet’s auto self-awareness back-off information received from the source node. This scheme will minimize the redundant transmission of packets and ensure reliable end-to-end data delivery. The concept of secure route formation is beyond the scope of this research paper. Interested readers should consult references such as [48,50] in the literature.

## 6. Performance Evaluation

In this section, we provide routing performance results that compare the ASAART, SHR and SSR protocols. Because our routing technique (ASAART) integrates the autonomous self-awareness and adaptive feature of routing path discovery and route repair into SHR, we use the Sensor Network Simulator and Emulator (SENSE) [36] for our performance evaluation. We consider both local and global network state information to compute the routing decision using the modified self-aware formula and backup strategies and efficient and reliable route repair technique as given in Section 5.

We performed five sets of simulations under a large and scalable sensor network with different simulated scenarios. In the first to third scenarios, we evaluate the network density test under a large and scalable sensor network. We consider a sensor network with 100 to 1000 nodes in step of 100 nodes. We vary the number of source nodes from 10 to 20 and 40 nodes in increasing network traffics. We evaluate protocol performance under increasing network density to observe the extent to which the proposed protocol is fault tolerant and resilient against errors with different network configurations. In the fourth and fifth scenarios, we evaluate the performance of the three protocols under transient and permanent node failure rates. In our simulation, we consider a 2000 × 2000 m^2^ terrain densely populated with 100 to 1000 nodes in step of 100 nodes, all which have a nominal transmission range of 250 m. We employed the free space propagation model in order to simulate the wireless medium [37]. We used the Constant-Bit-Rate (CBR) model to simulate the bi-directional traffic between the sources and destinations. The simulation at each scenario was performed at least five times with a different number of seeds to ensure accurate and reliable measurements and avoid errors. The 95% confidence intervals are shown in the figures.

### 6.1. Simulation Environment

The purpose of the simulation is to analyze the performance of our proposed autonomous self-aware and adaptive fault tolerant routing technique (ASAART) compared with SHR and SSR protocols. This allows us to study WSN behavior in the event of faults associated with the sensor nodes and the network, and to determine the extent to which the proposed scheme is fault tolerant. SENSE is used for the simulation [36,42]. 

**Figure 3:**
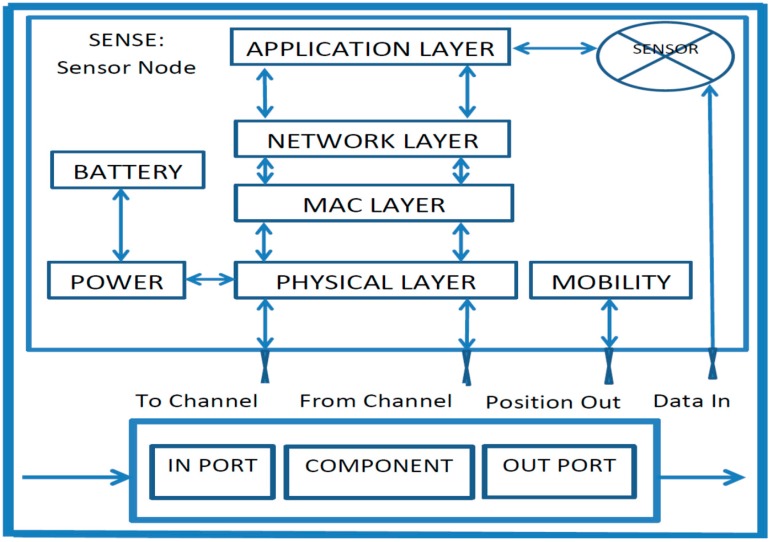
SENSE internal structure of sensor node.

The SENSE tool uses components that exchange information through ports. Two types of ports are considered the in-ports that are functional in nature and implement certain functionality similar to a function; in addition, an out-port acts as the function pointer that defines the functionality expected from other interfaces. The in-ports and out-port of the sensor node in SENSE are connected directly to the corresponding out-port and in-ports of the internal components. SENSE uses a simulation model based on the sensor node model depicted in Figure 3, which can be extended. With this model, it is possible to simulate several WSN scenarios. In SENSE, a sensor node is a composite component that features many smaller primitive components. Usually, a sensor has some layered network protocol components (PHY, MAC, NET, and APP), power and battery components related to power management, and other components, such as mobility and sensor [36,42].

### 6.2. Simulation Parameters

The following parameter values in Table 1 are used for the simulation. The values of these parameters are fixed and varied for different scenarios in order to provide efficient and reliable self-aware and adaptive fault tolerant routing techniques, and for better evaluation of the results.

**Table 1:** Simulation parameters.

Parameter(s)	Value
Network Size Terrain (m^2^)	2000 × 2000
Number of Nodes	100–1000 varied 600 nodes fixed
Number of Source Nodes	10, 20, 40 varied
Data Packet size (byte)	1000
Active Percentage	100%, 20% fixed 10%–60% varied
Active Cycle	100%, 20% fixed 10%–60% varied
Transmission Time (s)	0.002
Transmission Power (Tx) (Watt)	0.0290
Channel Model	Radio
Broadcast data packet	CBR
Slot Width (s)	0.0001
Simulation Time (s)	200
Scale Factor (s)	0.001
Traffic Direction	Bi-directional
Route Repair	SHR, ASAART (True) and SSR (False)

### 6.3. Performance Evaluation Metrics

In this subsection, we provide a brief explanation of the performance evaluation metrics used in this research paper. We evaluate the three routing protocols in terms of the sensor network density test and transient and permanent node failure rate tests. In order to have consistent and reliable routing performance evaluation, the five evaluation metrics listed below are used. Such metrics are widely accepted by the research community and are used for wireless sensor network performance evaluations [14,15,16,17,18,19,20,21,22,23,24,25,26,27,28,29,30,31,32,33,34,35,36,37,38,39,40,41,42,43,44,45,46,47,48,49,50,51,52,53,54,55,56,57,58,59]:

1. **Average End-to-End Data Packet Delivery (Success Rate):** This is the fraction or percentage of success among a number of attempts to transmit a packet.
2. **Average Packet End-to-End Delay:** This refers to the time required for a packet to be transmitted across a network from source to destination.
3. **Energy Consumption:** Defined as the ratio between the number of packets transmitted to the amount of energy consumed, where the amount of energy consumed is the difference between the initial energy before simulation to the energy used after the simulation. The unit is expressed in bits/joule.
4. **Average Number of Transmitted MAC Packets:** Total number of packets received at the MAC layer.
5. **Packet Drop Rate/Error Rate:** The Packet Error Rate (PER) is the number of incorrectly received data packets divided by the total number of received packets. A packet is declared incorrect if at least one bit is erroneous.

### 6.4. Simulation Results

In this sub-section, we provide the results of the simulation conducted based on five different simulated scenarios. These comprise of three different simulated scenarios for sensor network density test, as well as transient and permanent node failure rate tests. Details of the simulated scenarios are provided in the subsequent subsections.

#### 6.4.1. Sensor Network Density Test (First Simulated Scenario)

**Figure 4:**
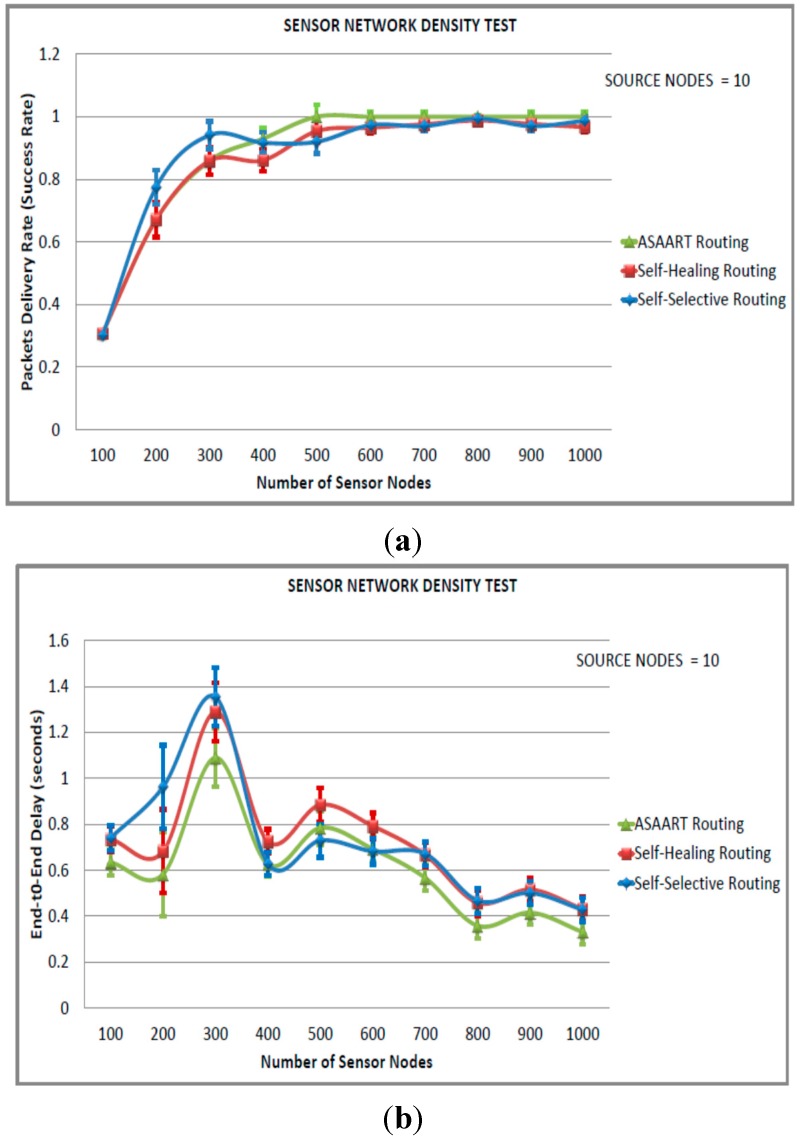

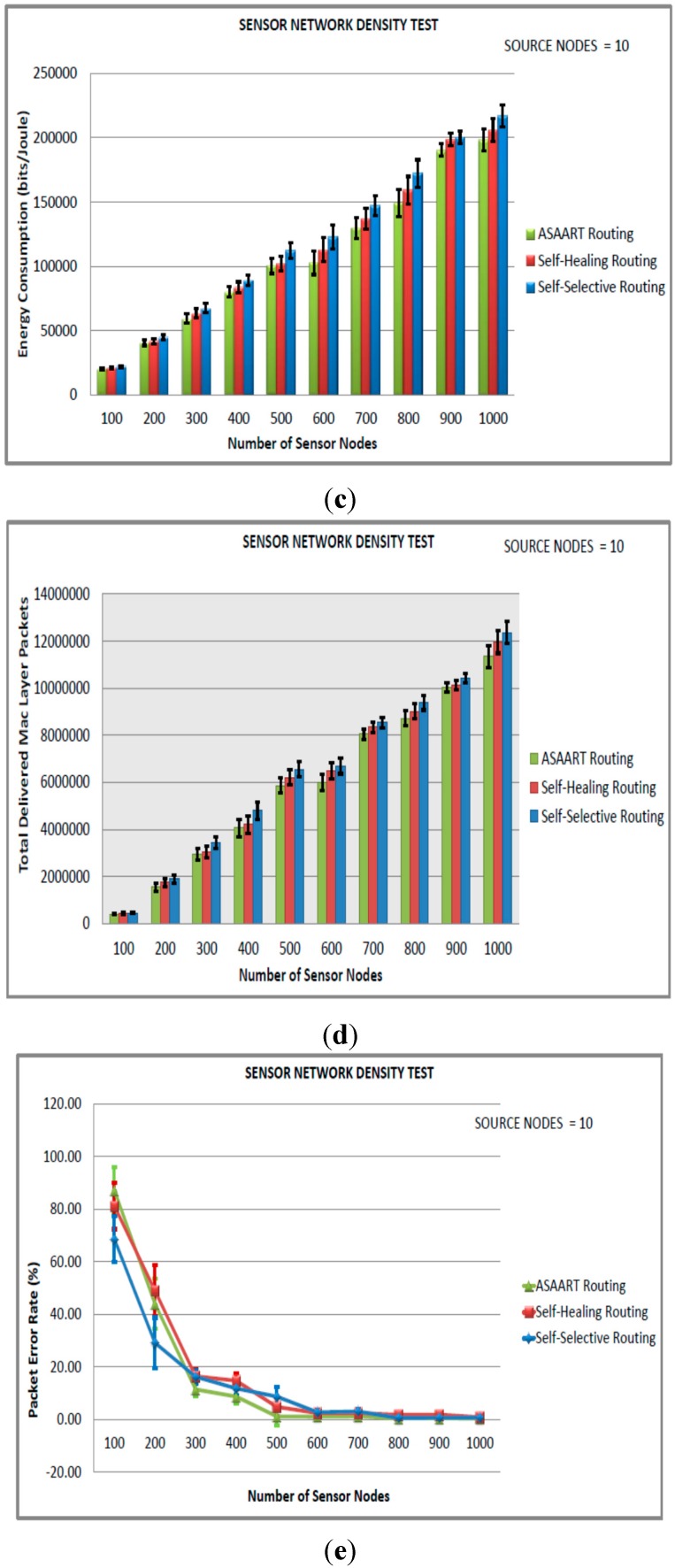
Sensor network density test (first simulated scenario) with source node = 10: (**a**) Average packet delivery rate (success rate); (**b**) End-to-end delay; (**c**) Energy consumption; (**d**) Transmitted MAC layer packets; (**e**) Packets error rate.

In this scenario, we set the number of source communicating nodes to ten, varied the network density from 100 to 1000 nodes in step of 100 nodes, and observed the routing performance. As observed from Figure 4a–e, the success rate drops to less than 100% between 100 to 1000 nodes by SSR and SHR protocols. However, ASAART maintain a 100% success rate between 500 to 1000 nodes compared with SSR and SHR protocols. This is attributed to the fewest number of communicating source nodes. There are fewer packets that reach the destination. However, the ASAART protocol slightly outperforms both SHR and SSR in terms of successful number of delivered network packets. 

In Figure 4b, ASAART end-to-end network delays show a slight reduction compared with SSR and SHR protocols, which means better reduction in the end-to-end delay. In Figure 4c,d, we can observe that ASAART protocol shows better energy consumption and a total number of reduced MAC layer packets delivery compared with the SSR and SHR protocols. This is attributed to its enhanced route repair mechanism that tries to control flooding at the MAC layer. In Figure 4e we can observe that ASAART packet error rate (PER) is lower at higher network densities compared with the SSR and SHR protocols. Showing a zero percent of error in packet delivery between the network of sizes 500 to 1000 nodes.

#### 6.4.2. Sensor Network Density Test (Second Simulated Scenario)

**Figure 5:**
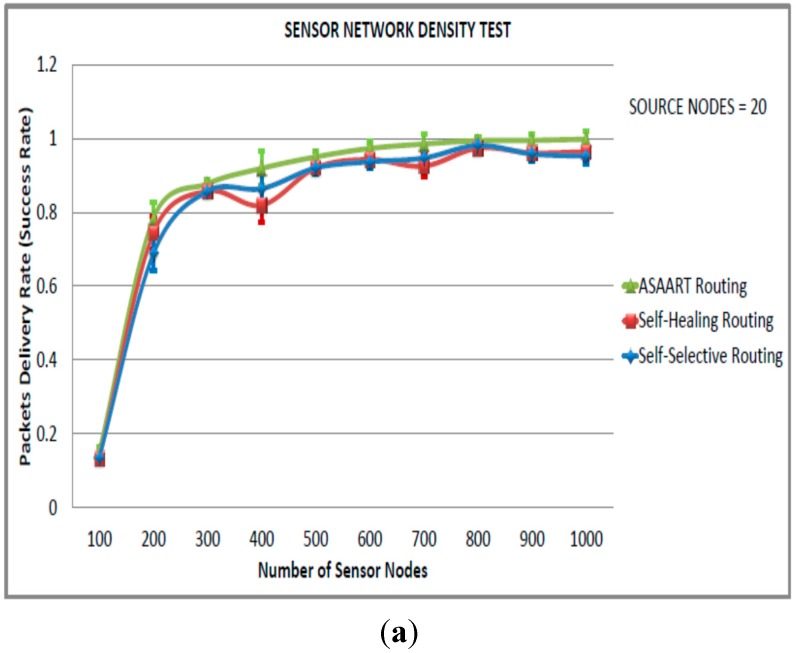

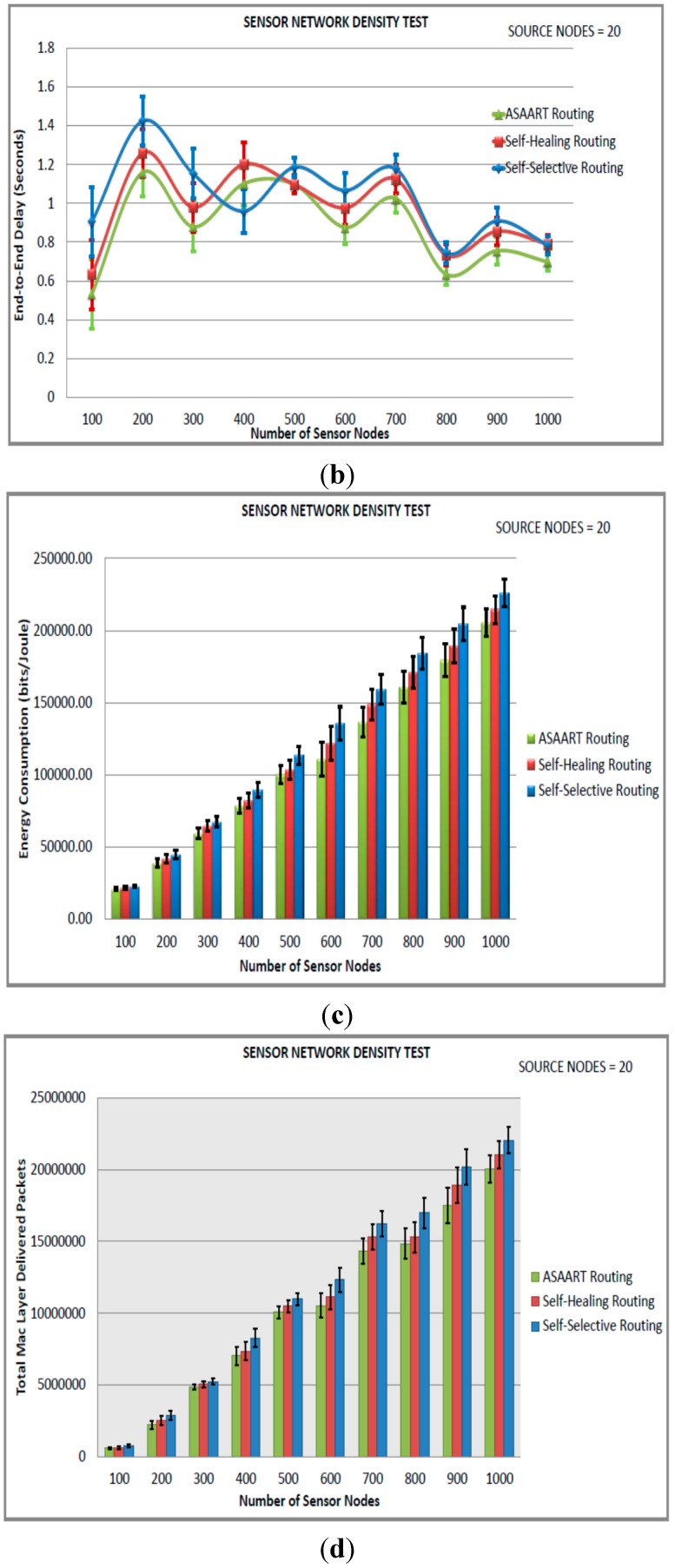

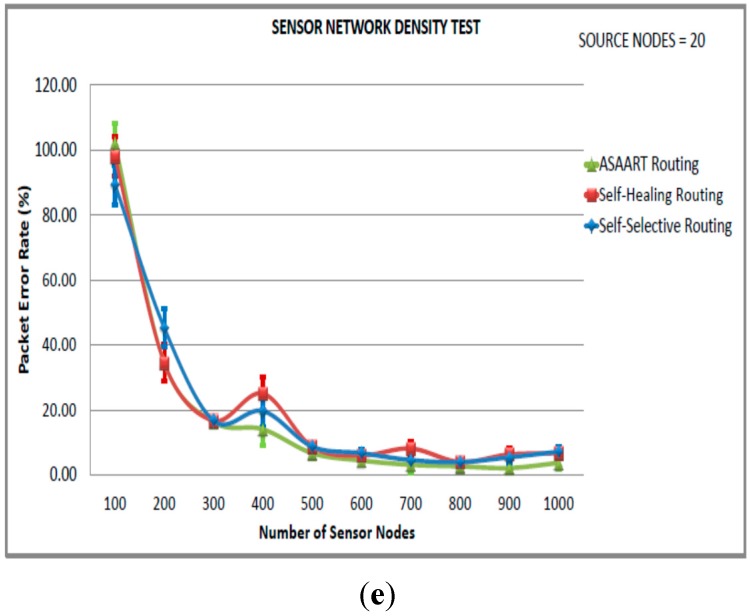
Sensor network density test (second simulated scenario) with source node = 20: (**a**) Average packet delivery rate (success rate); (**b**) End-to-end delay; (**c**) Energy consumption; (**d**) Transmitted MAC layer packets; (**e**) Packets error rate.

In this scenario, we fixed the number of source communicating nodes to 20 and varied the network size from 100 to 1000 nodes in step of 100 nodes. As can be observed from Figure 5a–e, the ASAART protocol provides the highest packet delivery rate or success rate compared with the SHR and SSR protocols. When the number of sensor nodes reaches 800 nodes, the success rate becomes stable with 100% packet delivery shown by ASAART protocol. However, at 400 node network size, SSR outperforms SHR protocol. This is because of its continuous back-off delay scheme that allows more flooding of network packets. However, at higher network density, both SSR and SHR protocols are competing achieving a stable success rate with over 95% delivered network packets.

We can observe that the end-to-end delay reduces significantly when the source nodes are set to 20 compared with the third simulated scenario with source equal to 40 nodes. We can also observe that between 100 to 400 nodes, the end-to-end delay is higher in all three routing protocols, with a maximum of 1.4 s exhibited by the SSR protocol. However, at a higher network density, *i.e.*, from 400 to 1000 nodes, the end-to-end delay reduces significantly with the ASAART protocol, showing better reduction in end-to-end delay.

From Figure 5c, we can observe that the energy consumption is minimal compared with the SSR and SHR protocols at different network densities. This is because there is a reduced number of delivered MAC layer packets that reduce network congestions and save energy consumption. In addition, in Figure 5e, we can observe that PER for the three routing protocols at higher network density becomes stable particularly, between 600 to 1000 network sizes. Any further increase in network density has no impact on performance.

#### 6.4.3. Sensor Network Density Test (Third Simulated Scenario)

**Figure 6:**
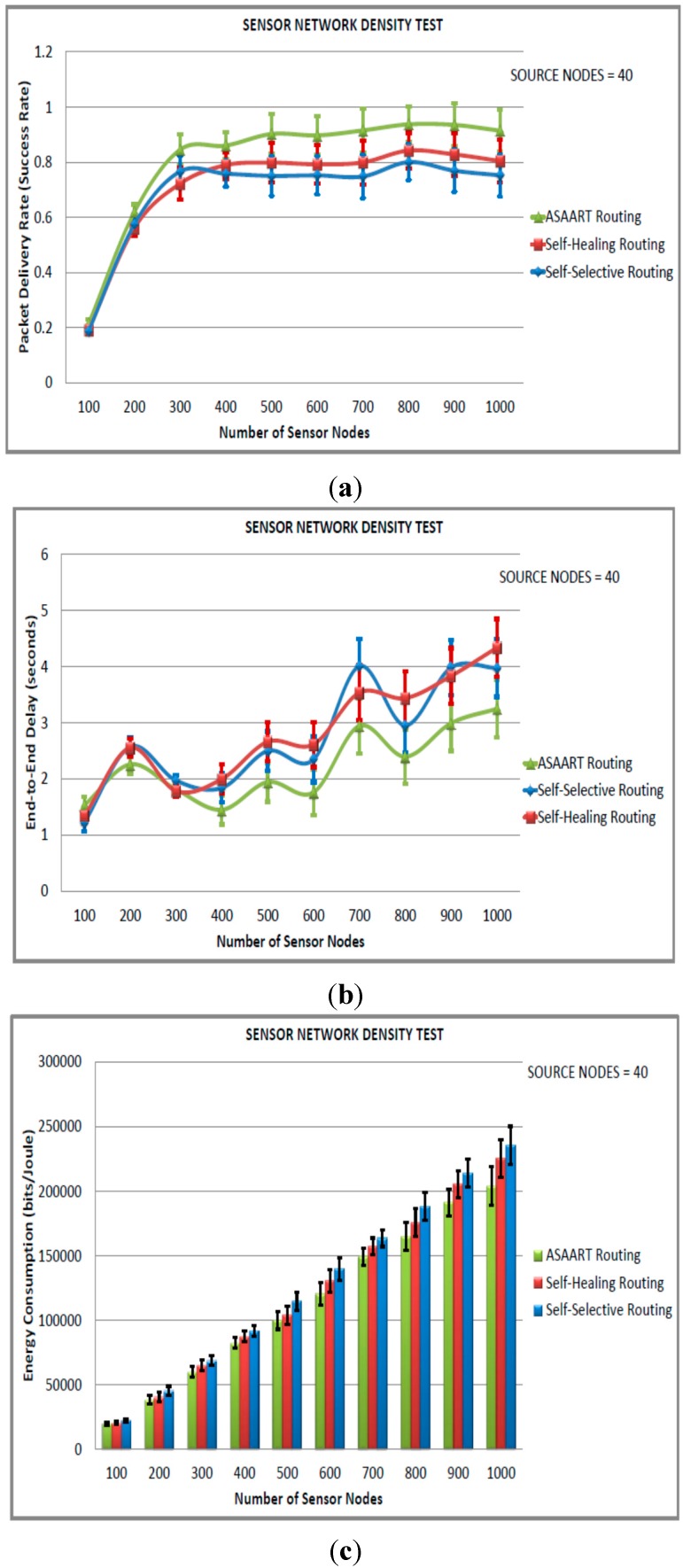

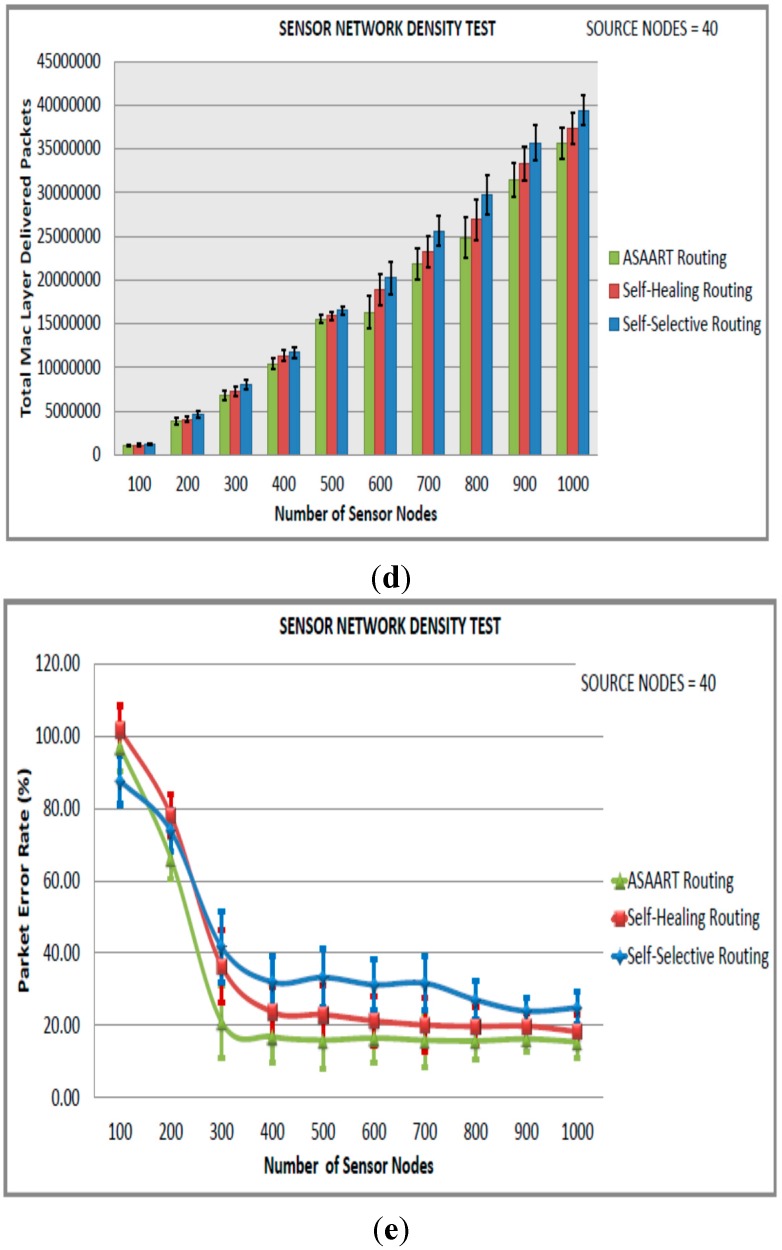
Sensor network density test (third simulated scenario) with source node = 40: (**a**) Average packet delivery rate (success rate); (**b**) End-to-end delay; (**c**) Energy consumption; (**d**) Transmitted MAC layer packets; (**e**) Packet error rate.

In the third scenario, we evaluated the impact of increasing the network density from 100 to 1000 in step of 100 nodes with 40 nodes designated as the source communicating nodes. The results of the simulation are illustrated in Figure 6a–e. As can be observed in the figures, increasing the network traffics and density causes too much traffic oscillations in both SSR and SHR, and decreases the success rate in the network, particularly between 100 to 300 node network sizes. However, at higher network density, the success rate of the delivered packets becomes stable, with SHR showing slightly higher packet success rate compared with SSR. Our ASAART protocol maintains higher packet success rates at different network densities. In addition, the time required to converge to minimal routing path is minimal, which makes the end-to-end delay lower than for SHR and SSR. At the higher network density, SHR spends too much time on topology maintenance, which causes its performance to reduce drastically compared with the ASAART technique. Despite the higher packet success rate and lower end-to-end delay exhibited by ASAART, it also uses minimum energy and reduced MAC layer packet delivery compared with SHR and SSR protocols. In Figure 6e, we can observe that PER for ASAART protocol is lower with a network of size between 200 to 1000 nodes compared with the SSR and SHR protocols. The SHR protocol competes against SSR by showing a slightly higher packet delivery ratio at higher network densities particularly between 400 to 1000 nodes. 

It is interesting to observe that when the network is scaled from 400 to 1000 nodes, PER becomes stable for the ASAART routing protocol. In conclusion, the ASAART protocol proves to be resilient against errors, and energy consumption and offers better routing performance compared with the SHR and SSR protocols in a scalable and high traffic sensor network.

#### 6.4.4. Transient Node Failure Test (Fourth Simulated Scenario)

**Figure 7:**
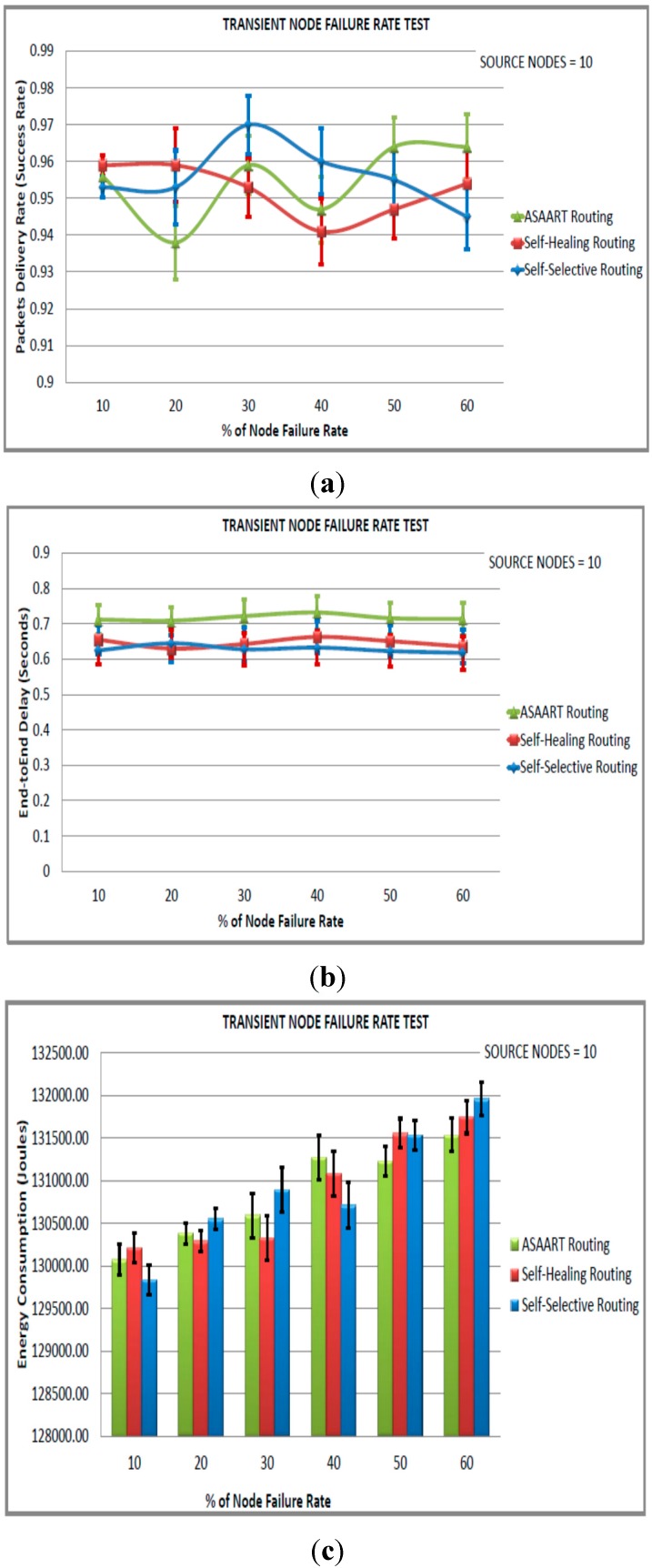

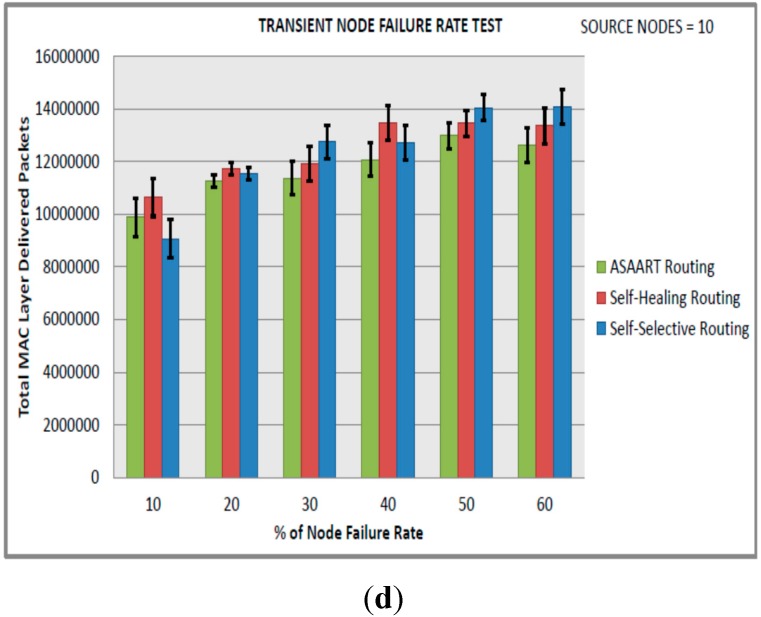
Transient node failure rate test (fourth simulated scenario) with source node = 10: (**a**) Average packet delivery rate (success rate); (**b**) End-to-end delay; (**c**) Energy consumption; (**d**) Transmitted MAC layer packets.

**Figure 8:**
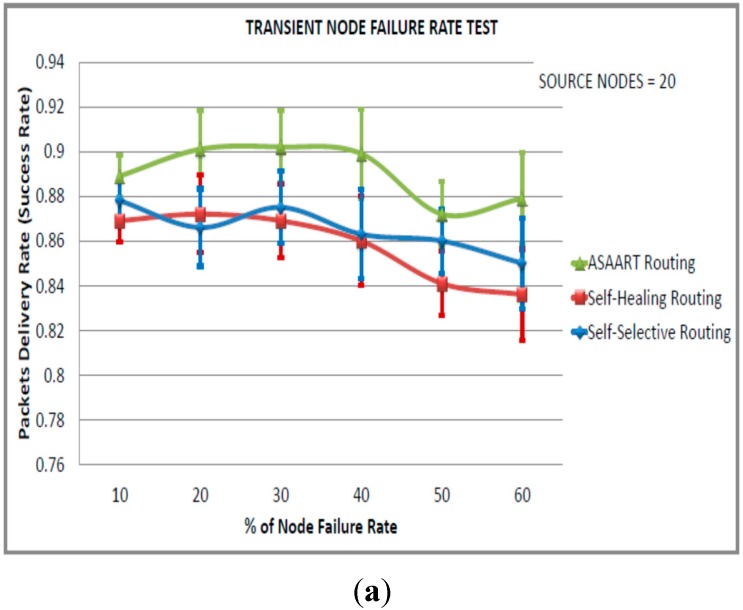

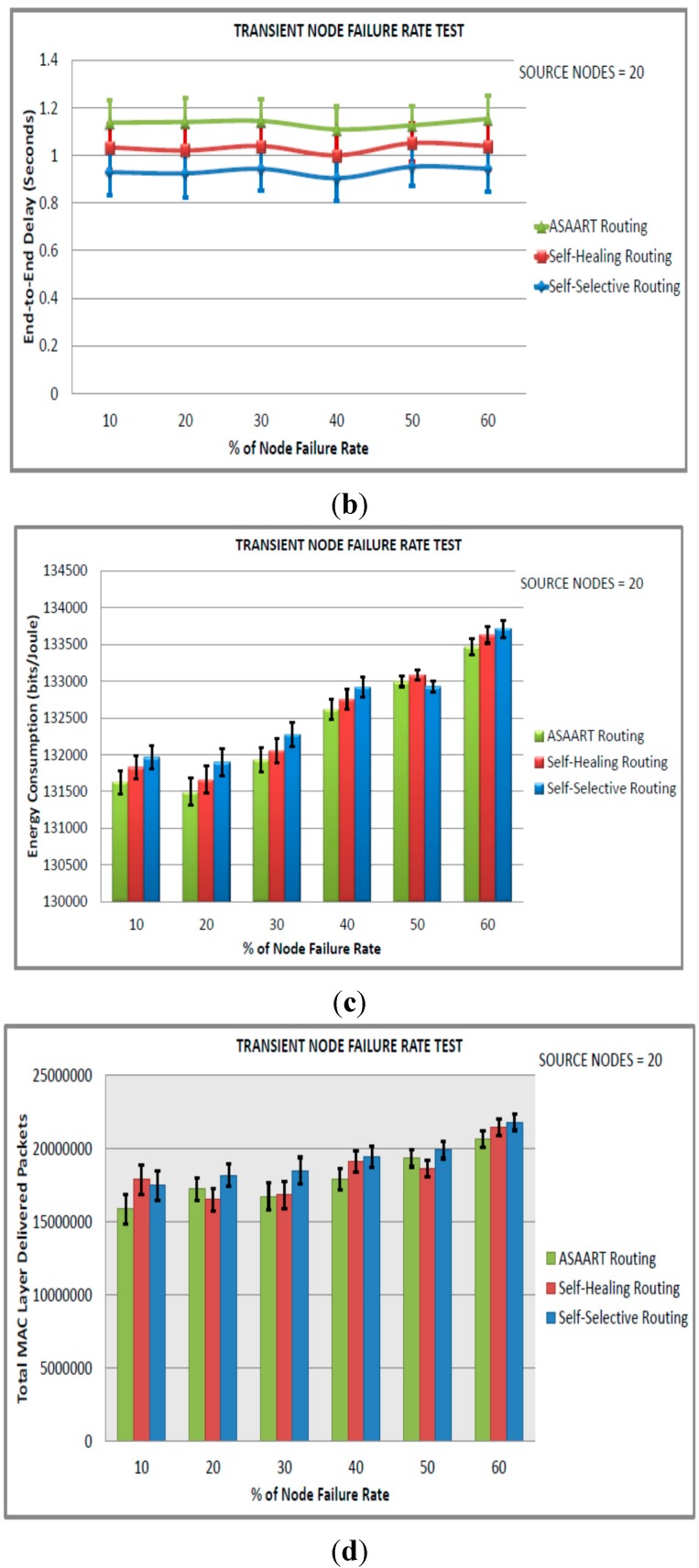
Transient node failure rate test (fourth simulated scenario) with source node = 20: (**a**) Average packet delivery rate (success rate); (**b**) End-to-end delay; (**c**) Energy consumption; (**d**) Transmitted MAC layer packets.

**Figure 9:**
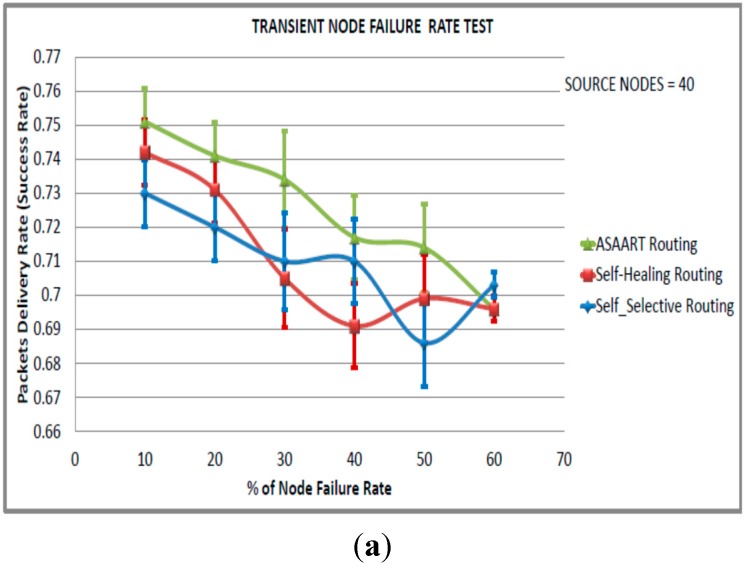

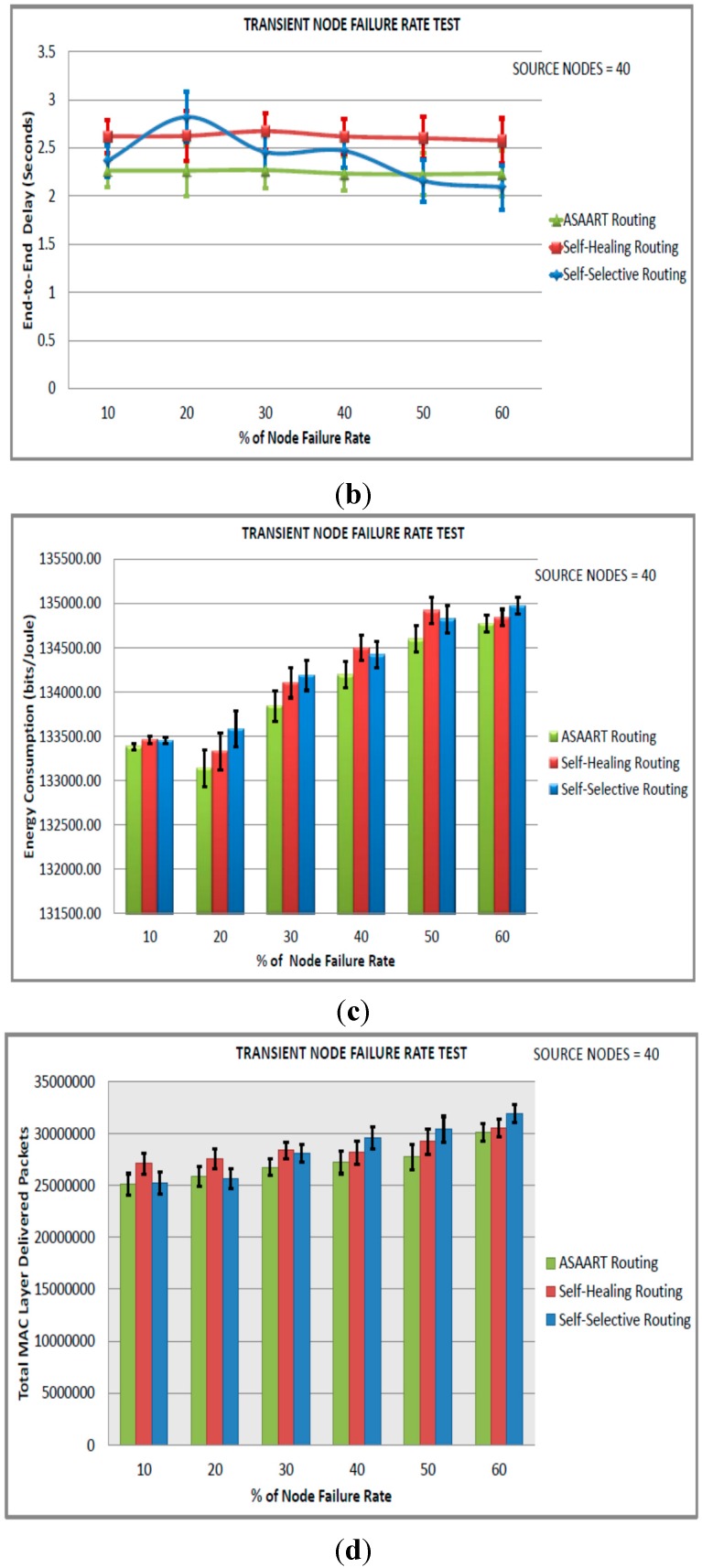
Transient node failure rate test (fourth simulated scenario) with source node = 40: (**a**) Average packet delivery rate (success rate); (**b**) End-to-end delay; (**c**) Energy consumption; (**d**) Transmitted MAC layer packets.

In this scenario, we consider a sensor network of size equal to 600 nodes. In order to simulate the transient node failure rate, we fixed the permanent node failure rate to 20% and varied the transient node failure rate from 10% to 60% in step of 10% in order to obtain routing protocol performance at different percentages of the transient node failure rate. We performed three sets of experiments with a different number of seeds, and varied the number of source communicating nodes in each scenario. The simulation results and 95% confidence intervals are shown in Figure 7, Figure 8 and Figure 9.

From Figure 7a–d, we can observe that the three routing protocols perform very well with a higher percentage of the packet delivery ratio. The three protocols achieve above 90% success rate because of less traffic congestion in the network. The ASAART performance drops to nearly 94% at 20% of the transient node failure rate. However, it slightly outperforms the SHR protocol when the transient node failure rate is between 30% and 60%, and the SSR protocol is between 50% and 60%. We observe that the ASAART protocol end-to-end delay is slightly higher than the SSR and SHR protocols. Nonetheless, this is negotiated by having the least number of MAC layer transmissions and energy consumption. ASAART attempts to minimize the retransmission of packets caused by flooding at the MAC layer, which makes it an energy efficient protocol.

Figure 8a–d show the performance of the three protocols with 20 nodes as the source communicating node under different transient node failure rates. We can observe that there is high routing performance in terms of the success rate exhibited by ASAART. The ASAART protocol achieves a success rate above 90% when the transient node failure rate is between 20% and 40%. However, when the failure rate is between 50% and 60%, the success rate drops to 87%. This is at the expense of slightly higher end-to-end delay compared with the SHR and SSR protocols.

We can also observe that the SSR protocol success rate slightly outperforms the SHR protocol. This is because of its prioritized continuous back-off delay strategy. However, SSR shows slightly higher MAC layer packet transmission because of flooding compared with the SHR and ASAART protocols. It is interesting to observe that the SSR protocol shows better reduction in the end-to-end delay compared with the ASAART and SHR protocols. From Figure 9a–d, we can observe that the ASAART protocol outperforms both the SHR and SSR protocols at a transient node failure rate from 10% to 50%, respectively. The SHR protocol success rate drops to 69% when the transient node failure rate is 40%. However, at 50%, it increases to 70%. The SSR protocol end-to-end packet delivery drops to 67% at 50% of the transient node failure rate. Surprisingly, however, it outperforms both the ASAART and SHR protocols when the transient node failure rate is 60%.

From Figure 9b, we can observe that ASAART end-to-end delay outperforms both the SHR and SSR protocols at a different percentage of the transient node failure rate. However, the SSR protocol outperforms ASAART at a transient node failure rate between 50% and 60%. The SHR protocol has the highest delay because of its route repairing mechanism. Too much time is spent repairing and retransmitting network packets. From Figure 9c–d, we can observe that ASAART uses slightly lower energy consumption and a reduced number of total MAC layer delivered packets compared with the SHR and SSR protocols. This reduces the network packet collision and contention, and saves energy. In conclusion, the ASAART technique demonstrates better fault tolerant capability in the presence of transient node failure rate compared with the SHR and SSR protocols, particularly when there is too much traffic congestion in the network.

#### 6.4.5. Permanent Node Failure Rate Test (Fifth Simulated Scenario)

**Figure 10:**
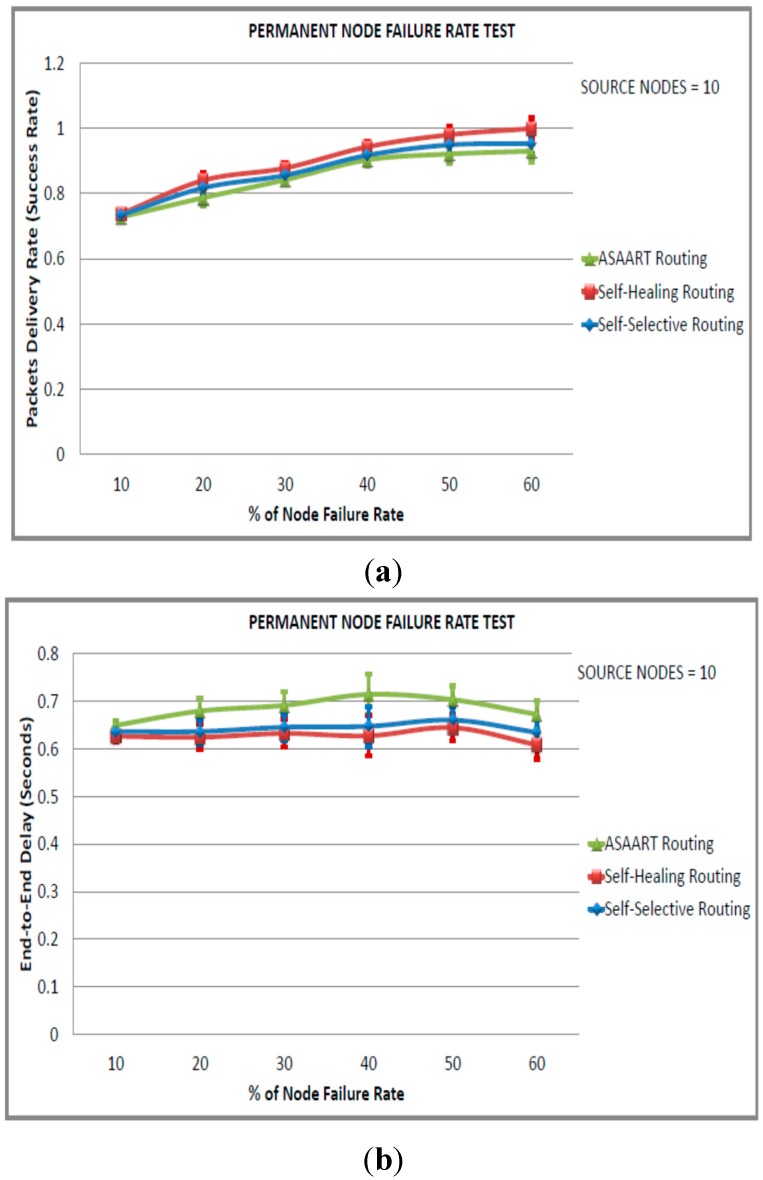

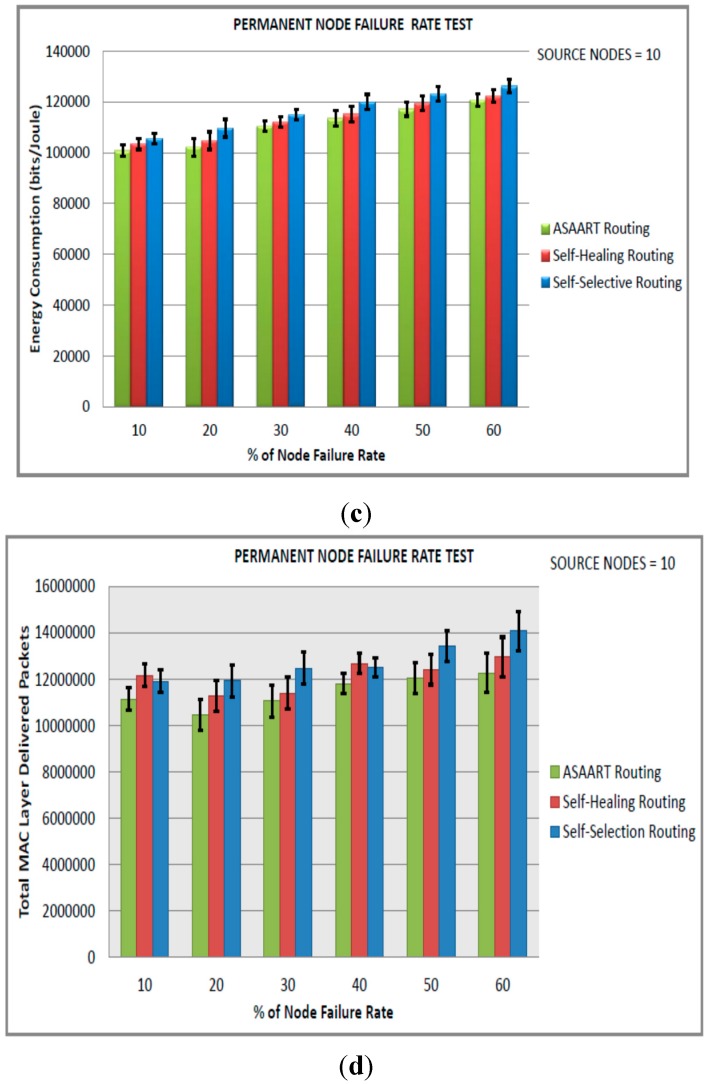
Permanent node failure rate test (fifth simulated scenario) with source node = 10: (**a**) Average packet delivery rate (success rate); (**b**) End-to-end delay; (**c**) Energy consumption; (**d**) Transmitted MAC layer packets.

**Figure 11:**
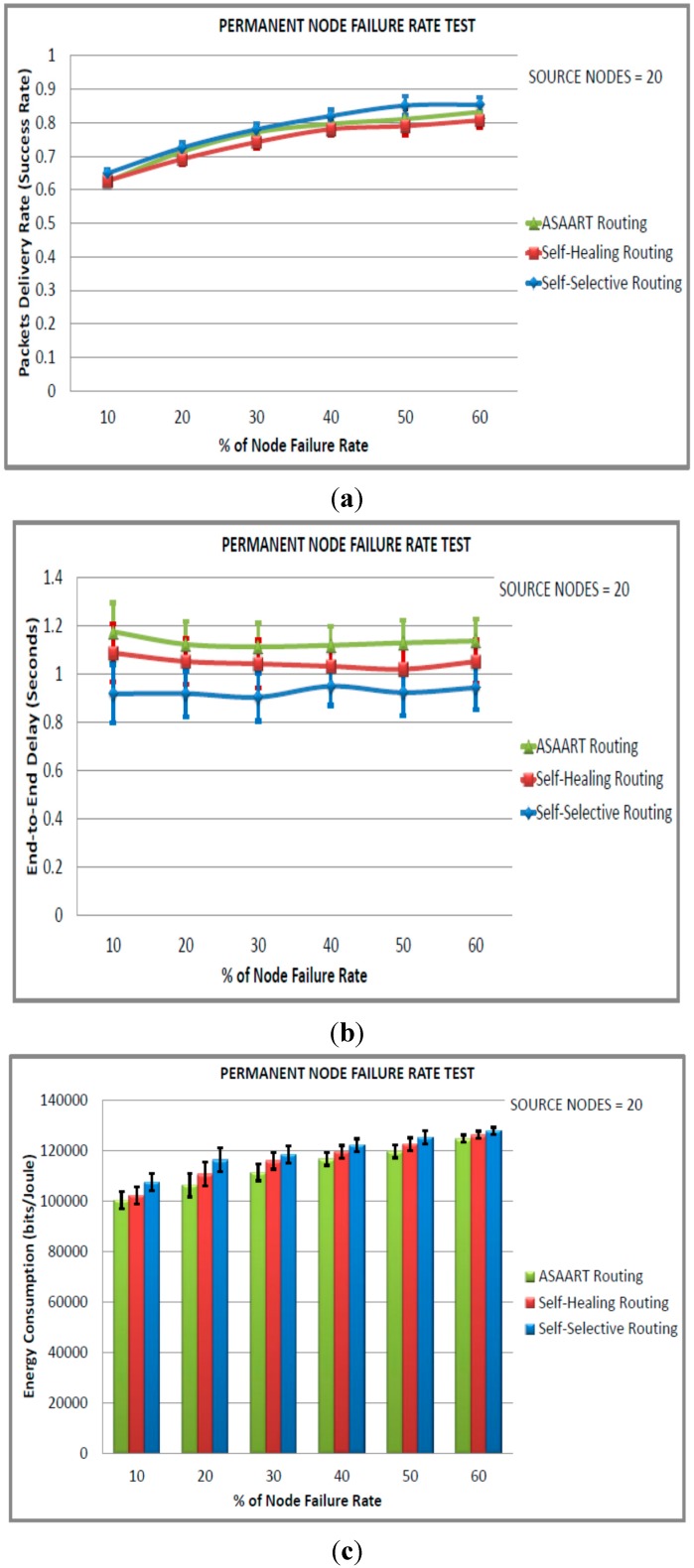

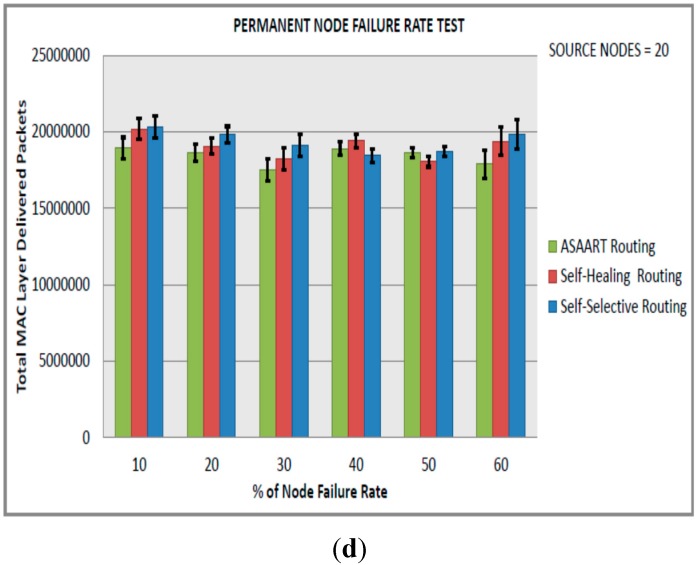
Permanent node failure rate test (fourth simulated scenario) with source node = 20: (**a**) Average packet delivery rate (success rate); (**b**) End-to-end delay; (**c**) Energy consumption; (**d**) Transmitted MAC layer packets.

**Figure 12:**
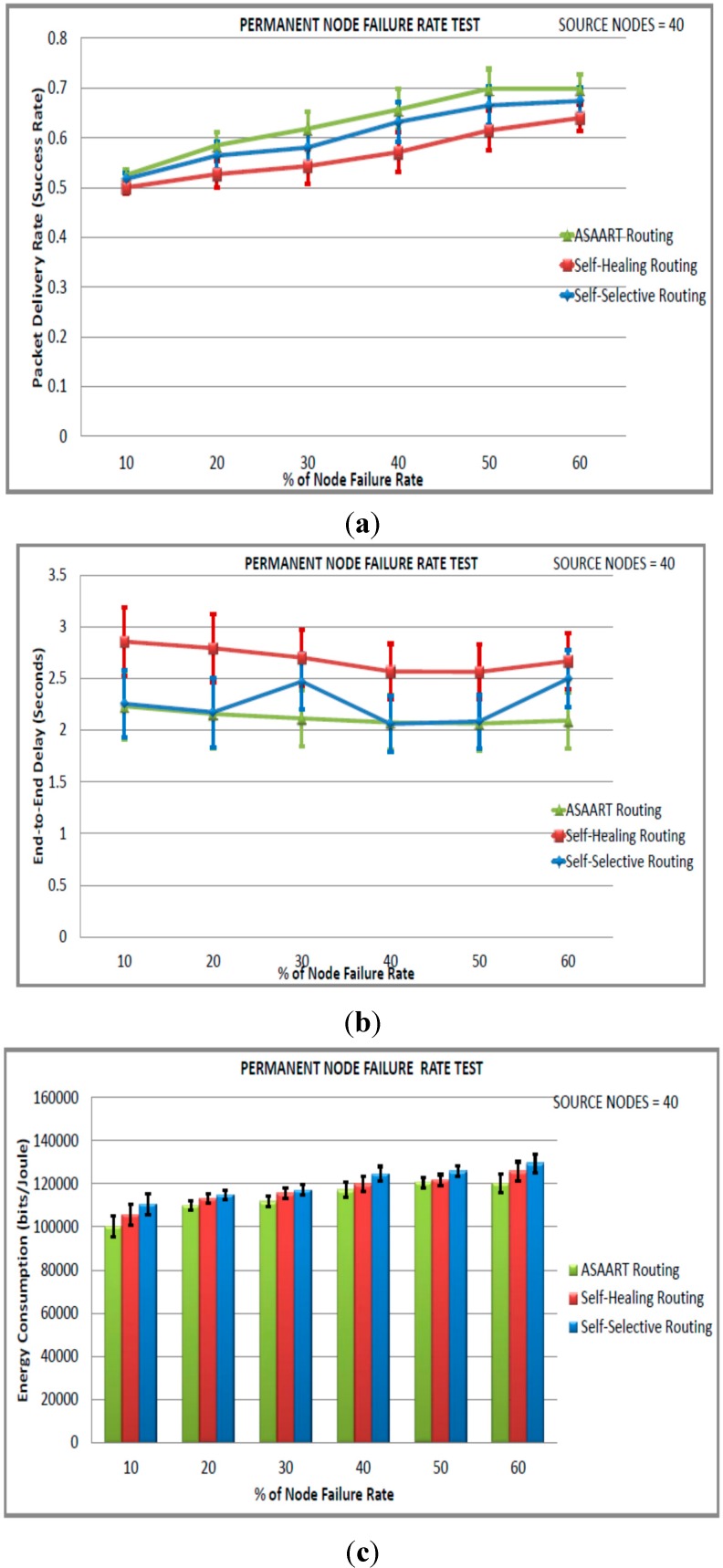

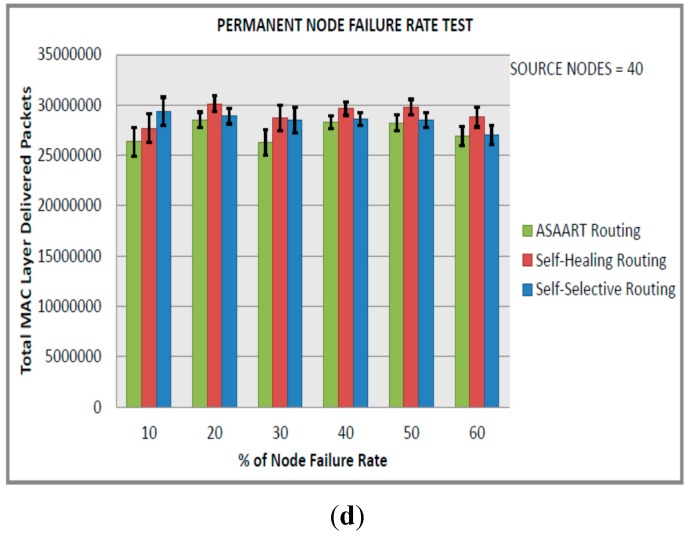
Permanent node failure rate test (fifth simulated scenario) with source node = 40: (**a**) Average packet delivery rate (success rate); (**b**) End-to-end delay; (**c**) Energy consumption; (**d**) Transmitted MAC layer packets.

In order to simulate the permanent node failure rate, we set each sensor node to fail for a fixed instance of time. The start of each sensor node failure rate was chosen randomly at the start of the simulation using the uniform distribution of the failure rate start time against the time of failure occurrences [24,40,41,51,52,53]. In this scenario, we consider a sensor network of size equal to 600 nodes. We set the transient link failure rate at 20%, and the source communicating nodes to be 10, 20, and 40 in order to ensure the protocol’s performance under high and low network traffics. We varied the permanent failure rate from 10% to 60% in step of 10% in order to obtain the routing performances at different percentages of the permanent node failure rate. We conducted three different sets of simulations with a different number of seeds, and varied the number of source communicating nodes in each simulated scenario. The results of the permanent node failure rate with 95% confidence intervals are illustrated in Figure 10, Figure 11 and Figure 12, which show the results of the permanent node failure rate performance evaluation of ASAART compared with the SHR and SSR protocols.

Figure 10a–d show the three routing protocols under permanent node failure rates with source communicating nodes of size equal to ten. Here we can observe that because of less traffic congestion in the network, the three protocols compete very closely in terms of the end-to-end packet delivery ratio. All three protocols attain 90% to 95% efficient packet delivery with a permanent node failure rate between 40% and 60%. The ASAART protocol shows slightly higher end-to-end delay and low packet delivery rate compared with the SHR and SSR protocols. However, this is compensated by having a lower MAC layer packet transmission and low energy consumption. ASAART achieves lower MAC layer transmission because of its flooding control technique that attempts to reduce the retransmission of redundant network packets.

From Figure 11a–d, we can observe an increase in the packet end-to-end delivery rate exhibited by the three routing protocols when the source communication nodes equal 20 compared with the source node being equal to 40 nodes. The three routing protocols achieve a success rate above 80% when the permanent node failure rate is between 40% and 60%. This is attributed to less network traffic, which helps reduce the packets’ contention and collision in the network. ASAART shows a higher end-to-end delay compared with the SHR and SSR protocols; this is because of its strategy of combined local and global network state information. However, ASAART exhibits better MAC layer packet transmission and energy efficiency compared with the SSR and SHR protocols.

In Figure 12, we can observe that the ASAART technique slightly outperforms both the SSR and SHR protocols. In Figure 12b, the SHR and SSR protocols exhibit higher end-to-end delay compared with the ASAART protocol. The ASAART protocol shows slightly lower end-to-end delay that remains almost stable when the permanent node failure rate is between 10% and 60%. ASAART uses slightly lower energy consumption compared with SSR and SHR, particularly at higher permanent node failure rates. This is because of its efficient route repair technique that ensures reliable end-to-end packet delivery. However, we can observe that ASAART uses the least number of delivered MAC layer packets at different percentages of the permanent node failure rate because of its efficient flooding control technique and faster routing decision that quickly converges to the minimum routing paths. In conclusion, ASAART protocol proves to be energy efficient and offer a high resiliency against faults and errors in the presence of permanent node failure rate and in highly congested and scalable network compared with SSR and SHR protocols.

## 7. Conclusions and Future Direction

In this paper, we proposed ASAART for wireless sensor networks to address the limitations of the SSR and SHR protocols. We integrated autonomous self-awareness and adaptive features into the SHR protocol to make it more resilient to faults and errors and energy efficiency for efficient and reliable routing of sensor data in wireless sensor networks. We achieved this by combining both continuous and prioritized slotted back-off delay, and multiple randomize function techniques to obtain local and global network state information for faster routing path formation and speedy convergence to the minimum routing path. We proposed a route repair technique for reliable transmission of sensor data in the presence of permanent and transient node failure rates, and efficient adaptation to simultaneous network topology changes. We conducted extensive simulations under five different scenarios. The simulation results showed that the ASAART technique performed better in terms of high resiliency to errors and failure and better routing performance and energy consumption compared with the SSR and SHR protocols in the presence of transient and permanent node failure rates and in a highly congested, faulty, and scalable sensor network. The proposed approach is energy efficient because it consumes less energy compared to the SSR and SHR protocols. Future research will address the issues of node mobility. In this case, we will integrate both local and global self-awareness and adaptive features for efficient and reliable cost table information update.

## References

[1] Santambrogio M.D., Hoffmann H., Eastep J., Agarwal A. Enabling Technologies for Self-Aware Adaptive Systems. Proceedings of the NASA/ESA Conference on Adaptive Hardware and Systems.

[2] Cheng S., Garlan D., Schmerl B., Steenkiste P. Software Architecture-based Adaptation for GRID Computing. Proceedings of the 11th International Symposium on High-Performance Distributed Computing (HPDC).

[3] Dini P. Internet GRID, Self-Adaptability and Beyond: Are we Ready. Proceedings of the 15th International Workshop on Database Expert Systems and Applications.

[4] Rengugadevi G., Sumithra M.G. (2012). Hierarchical Routing Protocols for Wireless Sensor Network—A Survey. Int. J. Smart Sens. Ad Hoc Netw..

[5] Braman A., Umapathi G.R. (2014). A Comparative Study on Advances in LEACH Routing Protocol for Wireless Sensor Networks: A survey. Int. J. Adv. Res. Comput. Commun. Eng..

[6] Kephart J.O., Chess D.M. (2003). The Vision of Autonomic Computing. Comput. J..

[7] HP Labs (2007). HP Open View Self-healing Services: Overview and Technical Introduction. User’s Guide, Software Version: 2.60.

[8] Breitgand D., Goldstein M., Henis E., Shehory O., Weinsberg Y. Panacea towards a self-healing development framework. Proceedings of the 10th IFIP/IEEE International Symposium on Integrated Network Management.

[9] Dustdar S., Dorn C., Li F., Baresi L., Cabri G., Pautasso C., Zambonelli F. A Roadmap towards Sustainable Self-aware Service Systems. Proceedings of the 2010 ICSE Workshop on Software Engineering for Adaptive and Self-Managing Systems.

[10] Birolini A. (1997). Quality and Reliability of Technical Systems: Theory, Practice and Management.

[11] Tanenbaum A.S., van Steen M. (2007). Distributed Systems: Principles and Paradigms.

[12] Souza L.M.S., Vogt H., Beigl M. (2007). A Survey on Fault Tolerance. Wireless Sensor Network.

[13] Dobson S., Denazis S., Fernández A., Gaïti D., Gelenbe E., Massacci F., Nixon P., Saffre F., Schmidt N., Zambonelli F. (2006). A survey of autonomic communications. ACM Trans. Auton. Adapt. Syst..

[14] Gelenbe E., Lent R. (2004). Power-aware ad hoc cognitive packet networks. Ad Hoc Netw..

[15] Han K., Luo J., Liu Y., Vasilakos A.V. (2013). Algorithm design for data communications in duty-cycled wireless sensor networks: A survey. IEEE Commun. Mag..

[16] Gelenbe E., Lent R., Nunez A. (2004). Self-aware networks and QoS. IEEE Proc..

[17] Zeng Y., Xiang K., Li D., Vasilakos A.V. (2013). Directional routing and scheduling for green vehicular delay tolerant networks. Wirel. Netw..

[18] Gelenbe E. (2009). Steps toward Self-aware Networks. Commun. ACM.

[19] Peng L., Song G., Shui Y., Athanasios V.V. (2014). Reliable Multicast with Pipelined Network Coding Using Opportunistic Feeding and Routing. IEEE Trans. Parallel Distrib. Syst..

[20] Gelenbe E., Gellman M., Lent R., Liu P., Su P. Autonomous smart routing for network QoS. Proceedings of the First International Conference on Autonomous Computing.

[21] Staddon J., Balfanz D., Durfee G. Efficient Tracing of Failed Nodes in Sensor Networks. Proceedings of the 1st ACM International Workshop on Wireless Sensor Networks and Applications.

[22] Tateson J., Roadknight C., Gonzalez A., Khan T., Fitz S., Henning I., Boyd N., Vincent C. Real world issues in deploying a wireless sensor network for oceanography. Proceedings of the Real-World Wireless Sensor Networks.

[23] Szewczyk R., Polastre J., Mainwaring A.M., Culler D.E. (2004). Lessons from a sensor network expedition. EWSN, LNCS 2920.

[24] Wasilewski K., Branch J.W., Lisee M., Szymanski B.K. (2007). Self-Healing Routing: A Study in Efficiency and Resiliency of Data Delivery in Wireless Sensor Networks. Proc. SPIE.

[25] Alec W., Tong T., Culler D. Taming the Underlying Challenges of Reliable Multi-Hop Routing in Sensor Networks. Proceedings of the 1st International Conference on Embedded Networked Sensor Systems.

[26] Robert D.P. Gradient Routing in Adhoc Networks. http://www.media.mit.edu/pia/Research/ESP/texts/poorieeepaper.pdf.

[27] Ye F., Zhang G., Lu S., Zhang L. (2005). Gradient Broadcast: A Robust Data Delivery Protocol for Large Scale Sensor Network. ACM Wirel. Netw. J..

[28] Hellsenbttel M., Braun T., Bernoulli T., Waelchli M. (2004). BLR: Beaconless Routing Algorithm for Mobile Adhoc Networks. J. Comput. Commun. Appl. Serv. Wirel. Netw..

[29] Zori M., Rao R.R. (2003). Geographic Random Forwarding (GeRaf) for Adhoc and Sensor Networks: Multihop Performance. IEEE Trans. Mob. Comput..

[30] Blum B., He T., Son S., Stankovic J.A. IGF: A Robust State-free Communication Protocol for Sensor Networks. http://libra.virginia.edu/catalog/libraoa:1152.

[31] Perkins C.E., Bhagwat P. Highly Dynamic Destination-Sequenced Distance Vector Routing (DSDV) for Mobile Computers. Proceedings of the ACM SIGCOMM’94.

[32] Bourndenas T., Wood D., Zerfos P., Bergamschi F., Sloman M. Self-Adaptive Routing Routing in Multi-hop Sensor Networks. Proceedings of the 2011 7th International Conference on Network and Service Mgt. (CNSM).

[33] Hu F., Hao Q. (2013). Intelligent Sensor Networks: The Integration of Sensor Networks, Signal Processing and Machine Learning.

[34] Krishnamachari B., Iyengar S. (2004). Distributed Bayesian Algorithms for Fault-Tolerant Event Region Detection in Wireless Sensor Networks. IEEE Trans. Comput..

[35] Park D.S. (2013). Fault Tolerance and Energy Consumption Scheme of a Wireless Sensor Network. Int. J. Distrib. Sens. Netw..

[36] Chen G., Branch J.W., Pflug M., Zhu L., Szymanski B.K., Lisee M., Chen G., Yener B., Szymanski B.K. (2006). SENSE: A Wireless Sensor Network Simulator. Advances in Pervasive Computing and Networking 2005.

[37] Rappaport T.S. (2009). Wireless Communications Principles and Practice.

[38] Del-Valle-Soto C., Mex-perera C., Monroy R., Nulazo-flores J.A. (2015). On Routing Protocol Influence on the Resilience of Wireless Sensor Networks to Jamming Attacks. Sens. J..

[39] Del-Valle-Soto C., Mex-Perera C., Olmedo O., Orozco-Lugo A., Galván-Tejada G., Lara M. (2014). On the MAC/Network/Energy Performance Evaluation of Wireless Sensor Networks: Contrasting MPH, AODV, DSR and ZTR Routing Protocols. Sens. J..

[40] Gilbert G.C., Branch J.W., Szymanski B.K. (2006). A Self-Selection Technique for Flooding and Routing in Wireless Ad-Hoc Networks. J. Netw. Syst. Manag..

[41] Babbitt T.A., Morrell C., Szymanski B.K. (2008). Self-Selecting Reliable Paths for Wireless Sensor Network Routing. Comput. Commun. J..

[42] Chen G., Szymanski B.K. Component Oriented Simulation Architecture towards Interoperability and Interchangeability. Proceedings of the Winter Simulation Conference.

[43] Duarte P.B.F., Fadlullah Z.M., Vasilakos A.V., Kato N. (2012). On the Partially Overlapped Channel Assignment on Wireless Mesh Network Backbone: A Game Theoretic Approach. IEEE J. Sel. Areas Commun..

[44] Liang L., Yuning S., Haiyang Z., Huadong M., Vasilakos A.V. (2015). Physarum Optimization: A Biology-inspired Algorithm for the Steiner Tree Problem in Networks. IEEE Trans. Comput..

[45] Yao Y., Cao Q., Vasilakos A.V. (2015). EDAL: An Energy-Efficient, Delay-Aware, and Lifetime-Balancing Data Collection Protocol for Wireless Sensor Networks. IEEE/ACM Trans. Netw..

[46] Gelenbe E. (2007). A diffusion model for packet travel time in a random multihop medium. ACM Trans. Sens. Netw..

[47] Liu X., Luo J., Vasilakos A. Compressed data aggregation for energy efficient wireless sensor networks. Proceedings of the 2011 8th Annual IEEE Communication Society Conference on Sensor, Mesh and Ad Hoc Communications and Networks (SECON).

[48] Attar A., Tang H., Vasilakos A.V., Yu F.R. (2012). A Survey of Security Challenges in Cognitive Radio Networks: Solutions and Future Research Directions. IEEE Proc..

[49] Gelenbe E., Sakellari G., D’Arienzo M. (2008). Admission of QoS-aware users in a smart network. ACM Trans. Auton. Adapt. Syst..

[50] Hind A., Agarwal A. (2013). A Multipath Routing Approach for Secure and Reliable Data Delivery in Wireless Sensor Networks. Int. J. Distrib. Sens. Netw..

[51] Gelenbe E., Liu P. (2011). Cognitive and Self-Selective Routing for Sensor Networks. Comput. Manag. Sci..

[52] Gilbert G.C., Branch J.W., Szymanski B.K. Self-Selective Routing for Wireless Adhoc Networks. Proceedings of the IEEE International Conference on Wireless and Mobile Computing, Networking and Communication.

[53] Branch J.W., Lisee M., Szymanski B.K. SHR: Self-Healing Routing for Wireless Adhoc Sensor Networks. Proceedings of the International Symposium on Performance Evaluation of Computer and Telecommunication Systems; SPECTS’07.

[54] Johnson D., Maltz D., Broch J., Perkins C.E. (2001). DSR: The Dynamic Source Routing Protocol for Multihop Wireless Adhoc Networks. Ad Hoc Networking.

[55] Chokers I.D., Elizabeth M.B.R. AODV: Routing Protocol Implementation Design. Proceedings of the 24th International Conference of Distributed Computing Systems and Workshops. W7–EC (ICDCSW’04).

[56] Intanagonwiwat C., Govinda R., Estrin D. Directed Diffusion: A Scalable and Robust Communication Paradigm for Sensor Networks. Proceedings of the 6th Annual International Conference on Mobile Computing and Networking.

[57] Heinzelman W.R., Kulik J., Balakrishnan H. Adaptive Protocols for Information Dissemination in Wireless Sensor Networks. Proceedings of the ACM MobiCom.

[58] Greedy Algorithm. http://www.encyclopediaofmath.org/index.php?title=Greedy_algorithm&oldid=34629.

[59] Akyildiz I.F., Su W., Sankarasubramaniam Y., Cayirci E. (2002). A survey on sensor networks. IEEE Commun. Mag..

